# Shining Light on Chitosan: A Review on the Usage of Chitosan for Photonics and Nanomaterials Research

**DOI:** 10.3390/ijms19061795

**Published:** 2018-06-17

**Authors:** Sreekar B. Marpu, Erin N. Benton

**Affiliations:** Department of Chemistry, University of North Texas, Denton, TX 76203, USA; erinbenton@my.unt.edu

**Keywords:** chitosan, luminescence, nanoparticles, bioimaging, sensors, surface plasmon resonance, quantum dots, plasmonic nanoparticles

## Abstract

Chitosan (CS) is a natural polymer derived from chitin that has found its usage both in research and commercial applications due to its unique solubility and chemical and biological attributes. The biocompatibility and biodegradability of CS have helped researchers identify its utility in the delivery of therapeutic agents, tissue engineering, wound healing, and more. Industrial applications include cosmetic and personal care products, wastewater treatment, and corrosion protection, to name a few. Many researchers have published numerous reviews outlining the physical and chemical properties of CS, as well as its use for many of the above-mentioned applications. Recently, the cationic polyelectrolyte nature of CS was found to be advantageous for stabilizing fascinating photonic materials including plasmonic nanoparticles (e.g., gold and silver), semiconductor nanoparticles (e.g., zinc oxide, cadmium sulfide), fluorescent organic dyes (e.g., fluorescein isothiocyanate (FITC)), luminescent transitional and lanthanide complexes (e.g., Au(I) and Ru(II), and Eu(III)). These photonic systems have been extensively investigated for their usage in antimicrobial, wound healing, diagnostics, sensing, and imaging applications. Highlighted in this review are the different works involving some of the above-mentioned molecular-nano systems that are prepared or stabilized using the CS polymer. The advantages and the role of the CS for synthesizing and stabilizing the above-mentioned optically active materials have been illustrated.

## 1. Introduction

With the ever-increasing demands of a rising human population, the competition for global resources is becoming even more dire On the one hand, high-performance materials are required for advancements in defense, space exploration, and biomedical research. On the other hand, environmental issues related to toxicity, sustainability, and cost-effectiveness need to be addressed. To overcome these increasing challenges, researchers around the world strive to produce technologies and materials that have positive impacts on the living conditions within society, which also minimize environmental impacts and production costs. The simplest solution is to use “smart” engineering, which uses “benign” materials obtained from renewable natural resources. This approach has become a key focus for researchers. Using polymers obtained from natural resources is one of the promising strategies employed by material-polymer scientists. CS and its derivatives fit well within this strategy. CS is nontoxic and has noteworthy physical and chemical properties that can be advantageous to various polymer-related technologies and products. For that reason, CS has been an attractive alternative for various research and commercial products [1]. CS, which is derived from chitin, is one of the most abundant polymers found in nature [2,3]. For example, chitin is one of the main structural components of an invertebrate’s exoskeleton and of the cell walls of fungi [2,3,4]. Chitin is a linear copolymer composed of glucosamine and *N*-acetylglucosamine units [2,3]. Producing CS from chitin involves protein and calcium salt removal and deacetylation with concentrated NaOH [2]. The addition of concentrated base causes hydrolysis of the aminoacetyl groups [2]. The extent of deacetylation can vary from 70–95%. When chitin is deacetylated to about 50% of the free amine form, it is referred to as CS [1]. The characteristics of CS are largely influenced by its composition and molecular weight [2]. Therefore, it is important to characterize the ratio between the glucosamine and *N*-acetylglucosamine units. This ratio can be determined by titration, nuclear magnetic resonance (NMR), infrared (IR), and ultraviolet (UV) spectroscopies [2]. Also, the molecular weight of CS ranges from 10,000–1,000,000 Da and can be calculated by viscosity and light scattering measurements [2,3,4]. Most types of CS are insoluble in organic solvents but can be dissolved in an acidic aqueous solution with pH < 6.5 [2,3,4]. Some commercial CS sources even contain a stoichiometric amount of acid to allow them to be soluble in water [2]. The p*K*_a_ of the amino group of the glucosamine is 6.5 [2]. Therefore, at pH less than ~6.5 the polymer is positively charged and can interact with other negatively charged surfaces resulting in various CS hybrids [2]. CS also has both amino and hydroxyl groups which can be used to alter its properties. Various examples of this are discussed in the next section [2].

The advantageous chemical and physical properties of organic polymers such as CS in combination with inorganic molecular materials and/or inorganic nanoscale building blocks create highly desirable products that possess the synergetic properties of both the organic polymer and the inorganic material. For example, such products would contain the flexibility, ductility, dielectricity, and processability of the organic polymer while also having the rigidity and thermal stability of the inorganic material. CS is highly desired for such applications. Among the relevant literature, Wang et al. [5] published a review detailing the usage of CS for developing CS-based inorganic nanocomposites including layered double hydroxide structures, metal nanocomposites, carbon nanomaterials, and metal oxide nanomaterials. Wang et al. [5] explained the advantage of combining a biocompatible, biodegradable, and nontoxic polymer with noble metals like gold, silver, and platinum, resulting in nanocomposites with antibacterial and catalytic properties. Compared to reviews published on the usage of CS for various other applications [6,7], reviews on the usage of CS for developing inorganic nanocomposites, optically active materials (e.g., plasmonic nanoparticles, semiconductor nanoparticles (also known as quantum dots), fluorescent organic dyes, and organometallic complexes containing Au(I), Ru(II), and Eu(III)) are very limited. To the best of our knowledge, the Wang et al. [5] review mentioned above is the only such review on this topic. However, the authors did not discuss CS hybrids containing luminescent materials. Many researchers in the field of optically active materials have selectively used CS polymer and its derivatives for stabilizing plasmonic nanoparticles, semiconductor nanoparticles, luminescent nanoparticles, and photoluminescent complexes. Due to the biologically benign nature of CS, such inorganic composite materials can be effectively used in the fields of optical sensors, biolabeling, bioimaging, and cancer research [8]. In this review paper, the many applications of CS will be discussed in brief with a focused discussion on the usage and applicability of CS for developing photoluminescent molecular nanomaterials and optically active colloidal, plasmonic, gold, and silver nanoparticles.

### 1.1. Selective Applications of Chitosan

#### 1.1.1. Antibacterial

With the growing world population there has been a significant increase in the use of antibiotics in the treatment of diseases. Unfortunately, there has been an overuse of these antibiotics that has resulted in the rise of antibiotic-resistant bacteria. Therefore, materials with non-conventional antibacterial systems that avoid the development of antibiotic-resistant “superbug” species like methicillin-resistant *Staphylococcus aureus* (MRSA) are in high demand. Inorganic nanomaterials of metals and metal oxides such as silver and zinc oxide (ZnO) have attracted more interest as antibiotic delivery systems than other inorganic nanomaterials. This is because of their stability, and the low probability that bacteria can develop resistance to these metal-based nanomaterials [9]. One of the most important features of CS is based on its polycationic nature and its antibacterial behavior. Being derived from naturally occurring chitin makes CS highly attractive compared to other antibacterial agents. CS inhibits growth of a wide variety of bacteria [10]. Compared to other established disinfectants, CS exhibits many advantages, such as killing a wider range of bacteria, higher killing rates, and a lower toxicity toward mammalian cells [10]. There are a few proposed mechanisms for the antibacterial activity of CS. The first mechanism involves the polycationic nature of CS interfering with the negatively charged residues of the macromolecules at the cell surface [11] of the bacteria. The other mechanism involves the binding of CS with DNA to inhibit mRNA synthesis [10,11]. Many researchers have concluded that various other factors also affect the antibacterial activity of CS including its molar mass, positive charge density, hydrophilicity/hydrophobicity, ionic strength, time, and temperature [12]. Hydrophilicity is also important in determining antibacterial activity, since most bacterial agents need water for exhibiting such a behavior [12]. This can make using CS difficult, since it has poor solubility in water [12]. Therefore, efforts to chemically modify CS to be more water-soluble are widening its use as an antibacterial agent [12]. The antibacterial behavior of CS is also found to be affected by pH and ionic strength [12]. Recently, numerous researchers have focused their attention on using CS as a stabilizing agent for the synthesis of metallic and metal-oxide nanoparticles with antibacterial properties. The CS-stabilized metal and metal-oxide hybrid nanoparticles are expected to be more stable, less toxic, and are expected to exhibit higher antibacterial efficiency due to the presence of the CS stabilizer with the metal or metal-oxide nanoparticles [9,13]. Of these, silver nanoparticles (AgNPs) and zinc oxide nanoparticles (ZnONPs) stabilized in CS have gained considerable interest [9,13]. AgNPs and ZnO NPs have excellent antibacterial, antifungal, antimicrobial, catalytic, electronic, and optical properties and therefore are widely used in various research activities [9,13] and commercial products. However, many believe that the biggest disadvantage of current nanoparticle synthesis methods is the reliance upon toxic/harsh chemical reducing and stabilizing agents [14]. This disadvantage is being overcome by the usage of the CS polymer. CS fits within the principles of “Green Synthesis”, which is favored over other chemical reduction methods because it avoids the use of harsh chemical reducing and stabilizing agents [14]. In these works, CS is shown to exhibit a triple role as a solvent medium, stabilizing medium, and reducing medium. Some researchers have extensively demonstrated the ability of a CS medium to act as both reducing and stabilizing agent to form different sized AgNPs and ZnONPs in the absence of any other reducing or stabilizing agents [15]. In summary, the environmentally friendly nature of CS and its biocompatibility have attracted much attention in the area of nanotechnology. Extensive details on the usage of CS for stabilizing these antibacterial nanoparticles (plasmonic silver nanoparticles) is discussed in later sections.

#### 1.1.2. Antioxidant

Oxidative stress is considered a critical factor in various degenerative diseases, as well as in the normal process of aging [16]. Reactive oxygen species (ROS), which are generated by normal metabolic processes or from exogenous factors and agents, result in damage to various essential biomolecules. Among different processes, accumulation of lipid peroxides due to peroxidation of membrane lipids is a well-established method for formation of ROS species [16]. The advantage of antioxidants is their ability to delay or prevent oxidation of essential biomolecules caused by ROS species [16]. Antioxidant mechanisms include scavenging ROS, activating detoxifying proteins, or preventing the generation of ROS [16]. Finding natural antioxidants is important, because they can protect the human body from free radicals and slow the progress of many chronic diseases [16]. Therefore, using functionalized CS to obtain polysaccharide-based compounds with antioxidant properties is of growing importance [17]. The oxidant scavenging activity of CS is due to the strong hydrogen-donating ability of CS, as well as its ability to chelate metal ions [18]. CS polymer has been shown to form very stable macromolecular radicals when reacted with certain oxygen species [18]. Overall, a higher concentration of low molecular weight CS has a positive influence on antioxidant activity [18]. Also, CS has been used as an effective antioxidant for human serum albumin (HSA), which is a major target of oxidative stress in uraemia and other vascular disorders [18]. In this study, the authors have concluded that low molecular weight CS prevented the formation of carbonyl and hydroperoxide groups in HSA protein [18]. A new approach involves incorporating flavonoids or other known antioxidants such as phenolic compounds, nitrogen compounds, and carotenoids into the CS structure [16,17,18]. For example, Guo et al. quaternized carboxymethyl CS derivatives, which showed better scavenging activity against hydroxyl radicals than regular CS [19]. This study demonstrated that the scavenging activity of carboxymethyl CS is directly related to the degree of quaternization, which emphasizes the influence of the positive charge on the scavenging activity against hydroxyl radicals [19]. Unfortunately, many methods of obtaining functionalized CS involve using toxic chemicals such as carbodiimide, ammonium persulfate, or formaldehyde [17]. Therefore, newer approaches involve enzymatic cross-linking, which is attractive due to an enzyme’s high specificity and environmental friendliness [17].

#### 1.1.3. Corrosion Protection

Corrosion is a common problem that significantly affects the properties of materials. Many materials exhibit resistance to corrosion due to the development of a stable oxide film over the surface. Coatings designed for corrosion protection must offer an effective physical barrier or form a stable oxide layer that protects the material’s surface against degradative or corrosive agents [20]. Therefore, corrosion protection requires the use of materials that are able to protect the underlying metal [20]. Additionally, the most desirable coatings must retain the ability to inhibit the corrosion process, even if the protective barrier is damaged [20]. Depending on the application of the coated material, including the number of layers applied, the final thickness of the coating system can range from a few up to several hundred micrometers [20]. According to Montemor, the most effective corrosion protection systems were based on the use of surface treatments, primers, and pigments based on chromates [20]. Due to the toxicity of these chromate-based treatments, a wide range of “green”, environmentally safe pigments and natural corrosion inhibitors have been developed [20]. CS is a potential candidate for application as a protective coating against corrosion of various substrates due to its unique environmental and biodegradable properties. This is based on its specific properties, which include good film-forming ability, superior adhesion to metallic surfaces, and versatility associated with the ease of chemical functionalization [21]. Unfortunately, CS has high-water permeability, which results in reduced barrier protective properties [21,22]. Therefore, to combat this problem, a self-healing effect is generally desired and is defined as the recovery of the coating integrity after damage occurs [22]. There was a study by Ghosh et al. on a self-healing polyurethane-based coating with oxetane-substituted CS precursor [23]. The authors explained that the four-membered oxetane rings open upon mechanical damage to the polymer network, which creates two reactive ends. Then, when exposed to ultraviolet light, the CS chain scission occurs, forming crosslinks with the reactive oxetane end, thus repairing the polymer coating network [23].

#### 1.1.4. Drug Delivery

One of the most promising and useful forms of CS are CS nanoparticles (CSNPs). Extensive reviews detailing the evaluation of the chemistry of CSNPs formation, and their impact on drug delivery systems for the treatment of diseases like cancer, are well documented [24,25]. Until now, nanoparticles approved by the United States Food and Drug Administration (USFDA) have been based on liposomes, polymers, or micelles [26]. Among various drug delivery platforms, nanoparticles-based delivery systems are known to exhibit several advantages such as target specific drug delivery, sustained release, enhanced solubility of hydrophobic drugs, increased concentration of the drug at the tumor site, and reducing immunogenicity [26]. Additionally, these nanoparticles are known to provide controlled release of chemotherapeutic agents in subcellular compartments such as endosomes and lysosomes, while not triggering the p-glycoprotein pump (known to promote multidrug resistance by expelling drugs from tumor cells) [26]. Although, numerous therapeutic nanoparticles have been developed based on various polymers, maximizing therapeutic efficiency while minimizing the amount of the drug is only possible using a targeted nanoparticles technique [26]. CS nanoparticles have also been investigated for the delivery of chemotherapeutic agents and cancer imaging agents [26]. Many CS nanoparticles have been used to deliver chemotherapeutic drugs to tumors via the enhanced permeability and retention effect [26]. Min et al. demonstrated the use of hydrophobically-modified glycol CS nanoparticles to deliver camptothecin (CPT) [27]. Insoluble CPT was encapsulated in the glycol CS nanoparticles with a loading efficiency above 80% [27]. Kim et al. also reported multifunctional glycol CS nanoparticles for cancer theragnosis [28]. These nanoparticles showed promise in terms of stability in serum, deformability, and rapid uptake by tumor cells [28]. Some of these CS nanoparticles were also loaded with well-established Near-Infrared Fluorescent (NIRF) dyes like Cy 5.5 for bioimaging applications and with anticancer drugs [28].

CSNPs or microparticles are formed by crosslinking CS with negatively charged polyanionic molecules (e.g., methacrylic acid and polyacrylic acid) or polyanionic molecules (e.g., tripolyphosphate (TPP) and dextran sulfate). Dialdehydes like glyoxal and glutaraldehyde are also commonly used as crosslinking agents for making CS nanoparticles. Many approaches including spray drying and coacervation/precipitation have been demonstrated in the preparation of CS micro and nanoparticles. The formation of CS hydrogels or nanoparticles by polyelectrolyte complexation (PEC) is an interesting alternative that crosslinks polymer chains via electrostatic interactions [29]. The cationic nature of CS has been conveniently exploited for the development of PEC-based microspheres and nanoparticles. An added benefit of this technique is that there are no toxic by-products from this synthesis approach. CS also has the ability to gel upon contact with certain polyanions, through a process called “ionotropic gelation”. This gelation process, which is mediated by the polyanions, occurs due to the formation of inter and intra cross-linkages between or within polymer chains. Many groups have adopted this technique for making size-tunable CSNPs using TPP as a gelling agent. Experimentally, in this method the CSNPs are synthesized via the addition of a basic solution of TPP with an acidic solution of CS. Upon mixing the solutions, CSNPs are formed immediately through inter and intra molecular linkages created between TPP phosphates and CS amino groups. Engineering nanostructures from CS polymer under the most benign and facile conditions is highly desirable in order to take advantage of all the intrinsic biocompatible properties of CS polymer.

#### 1.1.5. Food Packaging/Preservation

The goal of food packaging is to prevent tampering and contamination from either a chemical or biological source [30]. The real success of plastics in the food packaging industry is due to their flexibility, strength, low weight, stability, impermeability, and ease of sterilization. These attributes keep food free of contamination and are instrumental in keeping food fresh [31]. The ability to extend the shelf life of food products using plastics has been reported for different foods using a variety of techniques, such as resealable portioned packs, anti-microbial agents, humidity control systems, and modified atmosphere packaging solutions [31]. The crucial problem with using plastics for packaging is the post-consumer waste, which makes up 63% of all plastic waste [31]. This large amount of plastic waste from the food packaging industry is due to the fact that many plastics are difficult to recycle. Specifically, it is estimated that less than 14% of plastic packaging materials are recyclable [31]. CS can potentially help solve this issue, since the CS molecule has cationic groups along the backbone, granting it antimicrobial properties against bacteria, yeasts, and fungi (as discussed earlier) [31]. Also, its film-forming ability allows the production of membranes and coatings to act as a food preservative [31]. In addition, CS is biodegradable, biocompatible, non-toxic, renewable, and commercially available, all of which are highly desirable characteristics. Additionally, CS membranes are reported as being only semipermeable to gases presenting desirably low oxygen permeability, while maintaining a moderate water vapor barrier [31]. Both characteristics are essential for the preservation of certain foods. There have already been documented cases of CS being used to preserve food. Specifically, CS has been used to extend the shelf life of bread by retarding starch retrogradation and/or by inhibiting microbial growth [32]. The effect of a CS coating on the shelf life of baguettes has been studied by Ahn et al. [33]. The surface of the dough was coated with CS. The authors discovered that the baguette coated with CS showed less weight loss, hardness, and retrogradation compared to the experimental control during storage for 36 h [33]. The 36-hour shelf life of the CS treated baguette was extended by 24 hours compared with that of the 12-hour shelf life of the control. The same authors also showed that bread coated with CS had lower total bacterial counts and higher water content than that of the control after 8 days of storage. Mold growth was detected in the control after 4 days of storage, but was not detected in bread coated with CS throughout 8 days of storage. There is also ample evidence that CS coatings have the potential to prolong the storage life and control decay of fruits [34]. Strawberries are among the most perishable fruits and are vulnerable to physical injuries and fungal infection caused by *Botrytis cinerea* and *Rhizopus stolonifera* [34]. In one test by Ghaouth et al., strawberries were inoculated with a spore suspension of *Botrytis cinerea* or *Rhizopus stolonifera* and then dipped in CS solutions [34]. In both instances, the authors discovered that the CS coating significantly reduced the decay of strawberries. From the examples described above, it is very clear that CS can be effectively used as a food preservative to extend the shelf life of various food products [32]. However, due to the uncertainty in the sources of the CS used by the various researchers, there is a critical need to establish less expensive and more reliable analytical methods of CS characterization from a quality control standpoint [32]. Also, CS production process is costly, limiting its applicability in food applications. Simplification of CS production process is necessary to reduce production costs attributable to the current chemicals used in CS manufacturing and the associated processing/manufacturing time [32].

#### 1.1.6. Heavy Metal Removal/Water Treatment

Innovative technologies and materials are continuously being developed for treating industrial wastewater and contaminated water sources to remove soluble and insoluble impurities. This is a very important area of research in both developing and developed nations. Producing cheaper and effective technologies and materials that purify water while meeting regulatory standards is a continuous challenge. At present, municipal drinking water requires the removal of a wide variety of both physical and chemical pollutants, which include heavy metals, dyes, biodegradable waste, nitrates and phosphates, sediment, fluoride, hazardous and toxic chemicals, radioactive pollutants, pharmaceuticals, and personal products. Some of these pollutants are so toxic that even trace amounts of them will result in high volumes of contaminated water, which threatens human health and other living organisms [35]. Currently, different treatment methods are used to remove contaminants, which include coagulation/coprecipitation, oxidation/precipitation, ion exchange, adsorption, nanofiltration, reverse osmosis, bioremediation, and solvent extraction [35]. Among these techniques, adsorption is one of the most popular methods due to its simple operation, cost effectiveness, high efficiency, easy recovery, and regeneration capacity [35]. Activated carbon is currently the most commonly used adsorbent for heavy metal removal from aqueous solutions, although the focus of research is shifting toward adsorbents that are abundant, renewable, and biodegradable [36,37]. It has been established by many researchers that for heavy metal removal, an ideal adsorbent is expected to possess large surface area, high adsorption capacity, suitable pore size and volume, mechanical stability, compatibility, easy accessibility, ease of regeneration, cost effectiveness, environmental friendliness, simple processing procedures, and high selectively [35]. Some researchers have recently focused on developing materials with a chemically modified CS. Results indicated there is higher adsorption capacity for heavy metal ions than some conventional adsorbents due to the presence of the hydroxyl and amino functional groups on CS [35,36]. The higher adsorption potential of CS for heavy metals can be attributed to high water solubility, chemical reactivity of the hydroxyl and amino functional groups, and the flexible structure of the polymer chain [36]. Nevertheless, CS has some disadvantages such as low acid stability, poor mechanical strength, and low thermal stability, which restrict its field of applications [35]. Nonetheless, researchers are still working to apply additional physical and/or chemical modifications to further enhance its adsorption properties for metal ions [35,37].

#### 1.1.7. Tissue Engineering

Tissue engineering is an interdisciplinary science aiming to repair and replace tissues and organs [38]. The goal of tissue engineering is to restore, regenerate, maintain, or improve function in defective or lost tissue due to different disease conditions [39]. Scaffolds are three-dimensional structures that can be produced by synthetic polymers, natural polymers, or purely biological molecules, such as collagen, elastin, hyaluronic acid, and other extracellular matrix (ECM) molecules [38]. The scaffold must be able to mime the structure and biological function of natural ECM molecules in terms of both chemical composition and physical structure [38]. ECM molecules were originally known for their role in providing structural support to cells and as a location for cell migration [38]. Appropriate scaffolds for tissue engineering applications should be biodegradable, biocompatible, nontoxic, nonmutagenic, and nonimmunogenic [38]. Furthermore, scaffolds should be able to provide mechanical support and show desirable properties such as adhesion, proliferation, and differentiation of cells [38]. CS has features such as biocompatibility, biodegradability, and low toxicity, which makes it a viable candidate for a polymeric scaffold [38]. CS also has high blood compatibility and does not produce a large immune response [38]. However, the degree of deacetylation (DD) has an impact on the ability of CS to be a useful scaffold [39]. At a greater DD, the degradation of CS is slower and can last up to a few months [39]. The DD also plays a key role in cell adhesion and proliferation, but fortunately does not change the cytocompatibility of CS [39]. Another factor important for CS to be used as a scaffold is porosity. Porous scaffolds serve to provide support in tissue engineering, because they act as a platform and provide the necessary structure to physically guide the differentiation and proliferation of cells for tissue growth in vivo and in vitro [39]. CS has been extensively investigated for soft tissue replacement, because porous scaffolding can retain water and bioactive proteins in its polymeric structure [39]. Hydrated porous CS membranes have been shown to have at least three times the surface size and volume compared to non-porous CS membranes, but elasticity and resistance to traction are ten times smaller than non-porous membranes that were used as controls [39]. The porous CS structure can be stabilized by adding glutaraldehyde, polyethylene glycol, heparin, or collagen [39]. This allows the structure to become more traction resistant and to maintain elasticity [39]. CS membranes have a disadvantage when used for support in tissue engineering, because these membranes are very stiff and brittle, meaning they have low mechanical resistance [39]. In order to optimize resistance and elasticity, crosslinking agents are used with at least two functional reactive groups that allow for making bridges between polymeric chains. The following crosslinking agents have been demonstrated, namely: formaldehyde, epoxides reacting with polyethylene glycol, dialdehydes (glutaraldehyde and glyoxal), and starch [39].

## 2. Chitosan Polymer for Photonic Applications

In this review, the optically active, photonic systems that are stabilized and/or synthesized within CS are categorized into two types. The first type of optically active CS hybrids contains a luminescent agent and is labelled as a luminescent chitosan system. The second type of hybrid discussed contains optically active plasmonic nanoparticles and is labelled as a plasmonic metal nanoparticle-chitosan system. Examples of both types of optically active, photonic materials containing CS polymer are discussed in detail in the following sections.

### 2.1. Luminescent Chitosan Systems

**Figure 1:**
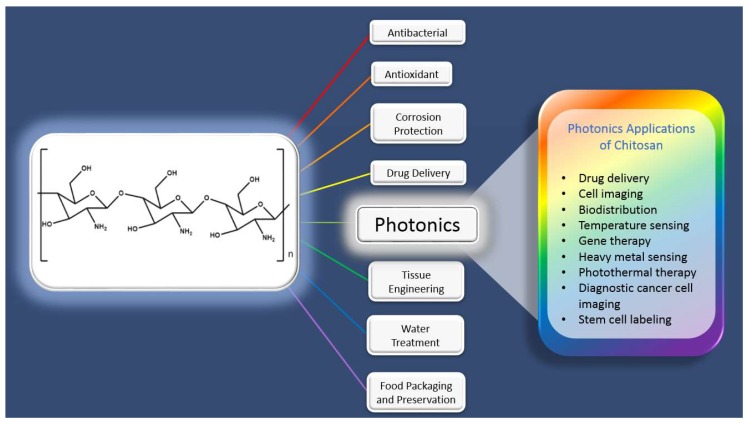
Schematic illustration showing different applications of chitosan (CS), highlighting the photonic applications of CS and its derivatives.

As shown in Figure 1, the usage of CS in research and commercial applications is widespread. Synthesizing luminescent CS and CS-based nanoparticles (which includes quantum dots, carbon dots, organometallic complexes, and aggregation-induced emission nanoparticles) is accomplished using various methods. These syntheses can be broadly classified into three types. In the first type, the luminescent nanocrystals or nanoparticles are capped with CS [40,41,42]. In the second type, the luminophores are directly doped into existing CS polymeric microspheres [43,44]. In the third type, CS is labelled using organic dyes like FITC by using a crosslinking agent. There has even been literature on synthesizing these nanoparticles using a combination of the synthesis methods mentioned above [40,41,42,43,44,45]. Additionally, our group has found a unique way of making CS nanoparticles by self-assembly of CS polymers using a negatively charged Au(I) molecular system. Electrostatic interactions between the Au(I) molecular system and the CS polymer resulted in the formation of size-tunable phosphorescent CSNPs [46]. In the following subsections, different forms of these optically active, photonic CS systems are discussed.

#### 2.1.1. Luminescent Chitosan Nanoparticles

Recent developments in nanoparticle technology have expanded applications of luminescent nanoparticles [47]. Specifically, CS-based fluorescent nanoparticles have attracted special attention for their biocompatibility, their ability to be conjugated to biological systems, and their compatibility with biological medium [8,45,48]. A recent review has listed important characteristics for a successful nanoparticle-based optical imaging agent, namely, in vitro and in vivo stability, resistance to metabolic disintegration, high quantum yield, large extinction coefficient, sufficient dispersibility in a biological environment, and a nontoxic nature of the contrast agent. Based on these requirements, the following is hypothesized: Highly desirable hybrid nanostructure [8,49] would be formed by stabilizing imaging agents in aqueous solution encapsulated with polymeric materials that can form nanoparticles, and possess reactive functional groups for further bio-conjugation. Therefore, CSNPs containing a luminescent agent (referred to as luminescent CS nanoparticles) are very attractive candidates, especially for in vitro drug delivery, cell imaging, biodistribution, diagnostic imaging of cancer cells, labeling of stem cells, imaging of pathogenic cells, temperature sensors, killing cancer cells, and other biomedical purposes [8,45,48,50,51]. For all such applications, CSNPs are either loaded or cross-linked with a luminescent reagent that exhibits photoluminescence on excitation with UV or visible wavelength light.

**Figure 2:**
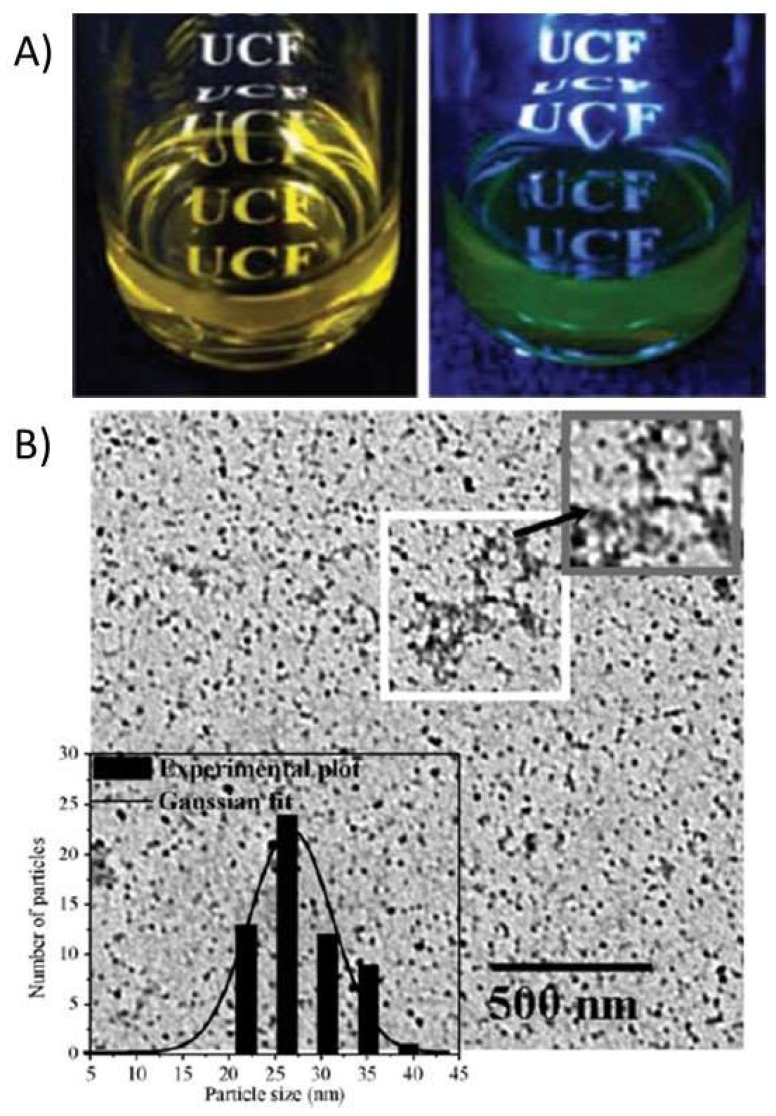
Demonstrates formation of FCSNPS using FITC. Daylight and emission pictures are shown in (**A**), and actual size and size distribution of the FCSNPs are shown in (**B**). Reprinted with permission from *Chem. Comm*. **2009**, 2347–2349, Copyright 2009, Royal Society of Chemistry [52].

**Figure 3:**
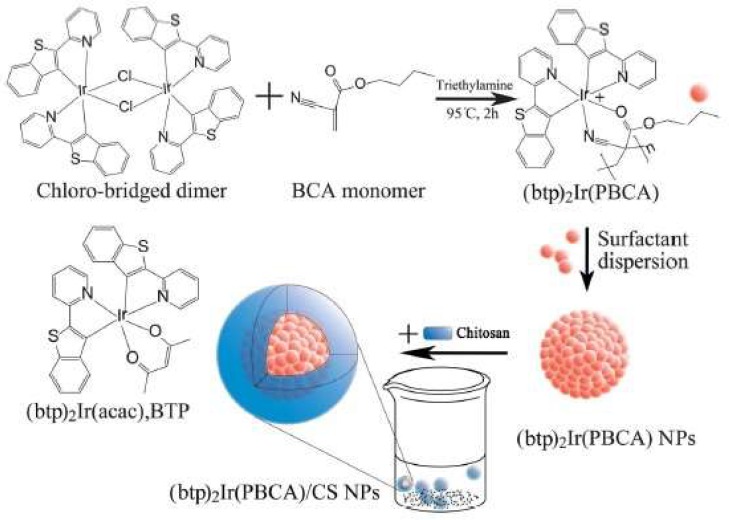
Demonstrates formation of phosphorescent CS nanoparticles using an Iridium-based organometallic complex. CS polymer is shown to encapsulate the phosphorescent molecular system during formation of phosphorescent nanoparticles. Reprinted with permission from *Nanoscale* **2013**, *5*, 12633–12644, Copyright 2013, Royal Society of Chemistry [55].

**Figure 4:**
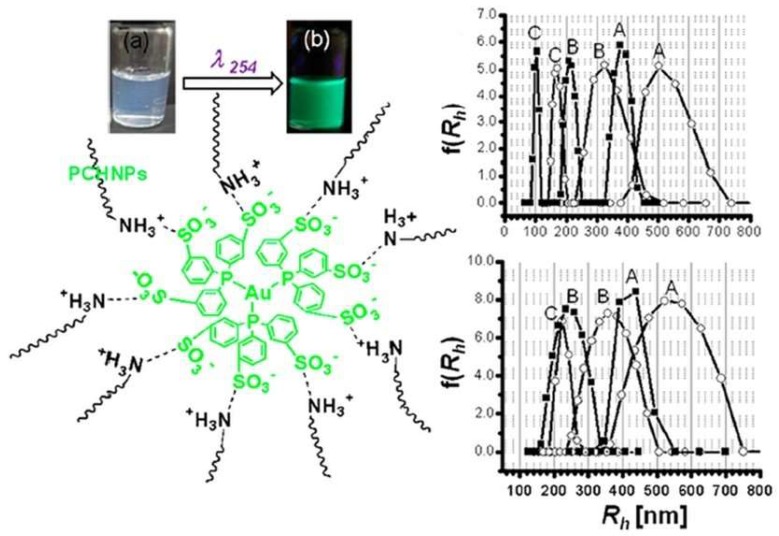
Schematic illustration for the formation of phosphorescent CS nanoparticles using Au(I) molecular system as a crosslinker. The light scattering data demonstrates the formation of size-tunable particles. Figure (**a**) CS nanoparticles in day light, (**b**) CS nanoparticles on excitation with UV lamp. A, B, C represents varying concentrations of Au(I) molecular system and CS polymer during synthesis of phosphorescent CS nanoparticles. Reprinted with permission from *J. Phys. Chem. C* **2015**, *119*, 12551–12561, Copyright 2015, American Chemical Society [46].

For labeling purposes, Ge et al. coated a magnetic core with a modified CS and covalently attached a fluorescent dye to the polymer. In this case, the cellular imaging labeling efficiency of hybrid nanoparticles with fluorescence and magnetic properties was evaluated. The incubation time and concentration of nanoparticles had a strong effect on labeling. The CS was modified with FITC by simple acid-amide chemistry [40]. Conjugation of fluorescent dyes like FITC to CS polymer is very common for various applications. Huang and coworkers have studied the cellular uptake of FITC conjugated nanoparticles to understand the internalization mechanism of fluorescent CSNPs in A549 cells [41]. The ability of CS to act as a capping agent is extensively utilized, especially with toxic, heavy metal-containing quantum dot nanoparticles. In order to render these quantum dots water soluble, chemically stable, and biologically compatible, and to avoid the leaching of toxic heavy metals, CS is utilized [42,43]. In some cases, researchers use CS to encapsulate the heavy metal-based systems and then utilize the functional groups on CS for further crosslinking with fluorescent dyes. Work by Tian et al. has demonstrated a similar approach for making a multi-functional monohybrid system. The CaF_2_:Eu nanoparticles are first stabilized within CS polymer via its capping ability, followed by utilizing the amine groups on CS for crosslinking with biomolecules and proteins like Bovine Serum Albumin (BSA) [44]. In some cases, the existing nanoparticles or nanoclusters are self-assembled using modified CS systems [51] or crosslinked with CS using selective crosslinking agents. Other works include studies detailing the synthesis of fluorescent CS nanoparticles using FITC-labeled CS. These studies employed a multistep microemulsion technique that included the non-luminescent cross-linkers 1-ethyl-3-(3-dimethylaminopropyl)carbodiimide hydrochloride (EDC.HCl) and tartaric acid [52,53]. In the other cases, the toxicity issue of quantum dots was successively overcome by capping the quantum dots with CS polymer [50]. Also, in the case of luminescent lanthanide nanoparticles, the presence of CS has improved solubility and enhanced biocompatibility. For example, Wang and co-workers [54] reported capping of LaF_3_/Eu^3+^ nanocrystals with CS polymer acting as a surfactant to improve biocompatibility and aqueous solubility. Tabrizian et al. and Nie et al., on the other hand, have separately reported covalent bonding of acid functionalized InGaP–ZnS and CdSe–ZnS quantum dots with CS polymer by using EDC (1-ethyl-3-(3-dimethylaminopropyl) carbodiimide hydrochloride) as a crosslinking agent [43,55]. In one of these cases, by using heavy-metal containing fluorescent quantum dots enriched with CS, Tabrizian et al. were able to demonstrate deep-tissue imaging. Another characteristic feature of these systems is the near infrared emission at 670 nm that has the ability to penetrate tissue three times deeper compared to visible light. The cell viability demonstration in this work demonstrates the feasibility of CS-enriched, heavy-metal-containing fluorescent quantum dots for biological applications. In many of these cases, CS was used as a capping agent or surfactant to overcome the toxicity or insolubility of luminophores. In a different case, Santra et al. and Wu et al. separately showed the formation of fluorescent, CS-based nanoparticles starting with FITC-labelled CS, involving crosslinking with tripolyphosphate (TPP) or EDC/tartaric acid mixture [52]. Figure 2 demonstrates the formation of ultra-small, water-soluble, fluorescent chitosan nanoparticles (FCSNPs) by Santra et al. The figure shows very clearly the daylight and fluorescent images of luminescent CS nanoparticles that are synthesized using FITC dye. An interesting work by Zeng et al. has demonstrated the in vitro and in vivo tumor imaging using phosphorescent CS nanoparticles (Figure 3). The authors synthesized a hypoxia-sensitive coordination compound based on iridium, followed by formation of core-shell particles. They observed that compared to the naked dye, the dye/CS nanoparticles exhibited a significant decrease in phosphorescence lifetime, but the hypoxia sensitivity increased in the nanoparticle systems [56]. Although there are some electroluminescent-based Ruthenium/CS hybrids, the scope of this article is limited to photoluminescent systems. Different from the above-mentioned works, our group has utilized CS to synthesize phosphorescent CS systems. The phosphorescent CS systems are uniquely synthesized using luminescent Au(I) molecular systems as the emissive agent. In one of our studies, by using a polyanionic gold (I)-based molecular system, we demonstrated the formation of size-tunable phosphorescent CSNPs. Here, the emissive agent, Au(I), was observed to play a dual role: imparting luminescence and acting as crosslinking agent. In this work, our group observed that by selecting an appropriate doping/luminescence agent, usage of any extra crosslinking agent can be avoided. The emissive or luminescent agent will itself act as a crosslinker, resulting in the formation of size-tunable and highly stable phosphorescent CS nanostructures that are applicable to different biomedical scenarios (Figure 4). In the following sections, some of the most common luminescent systems such as quantum dots (e.g., cadmium selenide (CdSe), zinc sulfide (ZnS), and ZnONPs), metal nanoclusters (e.g., gold nanoclusters), organic dyes (e.g., FITC), aggregation-induced emission (AIE), lanthanide based chelates (e.g., Eu(III) and Tb(III) complexes), and transitional metal complexes (e.g., Au(I), Ru(II), and Ir(III) systems) are discussed in detail. 

#### 2.1.2. Quantum Dots

Today, bioimaging has evolved as a powerful tool in biological research because of its unique ability to provide chemical and physiological information regarding cells and tissues [57]. Fluorescence-based bioimaging techniques have been greatly encouraged due to several advantages, such as high sensitivity, high selectivity, diversity of use, and non-destructive nature of the imaging agent [57]. Current fluorescent dyes used for imaging and detection undergo a degradation process known as photobleaching that is irreversible and is known to severely hinder the imaging ability of the dyes [58]. Fluorescent quantum dots (QDs) (1–10 nm) are an attractive alternative for biological labelling because of their unique size-tunable fluorescence properties and excellent photostability [58,59]. The interesting optical properties of QDs are due to quantum confinement of electron motion. The quantization of energy levels resulting from quantum confinement of electrons results in emission, which is dependent upon the size of the QDs. QDs have robust fluorescence intensity with the advantage of a narrow emission and broad excitation wavelengths [58,59]. However, the main concern with QDs for biological imaging is their toxicity, since they are typically made from combinations of zinc(II), cadmium(II), selenide, and sulfide [58,60]. Biocompatible polymers like CS were introduced with the toxic QDs to improve hydrophilicity, stability, functionalization, and biocompatibility, making these CS coated QDs useful for biological imaging and sensing applications [58]. Incorporation or entrapment of QDs within a biocompatible polymer also enhances colloidal stability of QDs, and allows the possibility of loading other materials such as drugs for multitudinal applications [58]. In biological applications embedding QDs in a CS has also been known to enhance cellular uptake, while imparting fluorescent characteristics to the cells [58]. Sometimes CS films containing QDs were fabricated into sensor-based films for removal of heavy metal ions. In one of the works reported by Hussain et al., they fabricated luminescent ZnS QD CS films. In this work, the authors exchanged the Zn cation with Hg, Ag, and Pb, resulting in non-emissive HgS, Ag_2_S, and PbS QDs. The distinctive colors helped to identify these non-luminescent compounds. The detection limit for Hg^2+^ was recorded as 5 ppm. Here, the CS polymer’s ability to form films and interact with heavy metals was taken into consideration for doping the QDs [61].

#### 2.1.3. Carbon Dots

Carbon dots (CDs) were first reported in 2006, and are clusters of carbon atoms with diameters of typically 2 to 8 nm, but can also contain substantial fractions of oxygen, hydrogen, and nitrogen [60]. As opposed to the previously discussed QDs, CDs are biocompatible, environmentally friendly, and contain no heavy metal ions [62]. There are many materials used to cover the surface of the CDs, imparting high quantum yield, stability, and water solubility. Also, CDs can be conjugated with target molecules to expand their functionality [62]. The emission color of CDs can also be tuned to some extent by varying the experimental conditions of synthesis [60]. The excitation and emission spectra of CDs are very wide and usually extend from the UV to the red (650 nm) [60]. Several methods to prepare CDs have been reported [63]. One way is to etch a larger carbon structure onto individual CDs. This is accomplished using numerous methods including laser ablation of graphite; electrochemical oxidation of graphite and multi-walled carbon nanotubes; chemical oxidation of candle soot, natural gas, commercially activated carbon, and lampblack; chemical oxidation of oxide graphene; and by using silica spheres as nanoreactors [63]. Other synthesis approaches include chemical and thermal oxidation or microwave pyrolysis of carbonaceous compounds [63]. However, most of these synthesis methods require a strong acid and then further treatment with other compounds to improve water solubility and modify the photoluminescent properties [63]. Therefore, a new synthesis method has been developed for highly amino-functionalized fluorescent CDs by hydrothermal carbonization of CS, in which neither a strong acid nor surface passivation reagent is needed [63]. The synthesis process occurs in aqueous solution and has the advantage of being very cheap and environmentally friendly [63].

#### 2.1.4. Aggregation-Induced Emission Nanoparticles

Strategies for fabricating luminescent probes based on aggregation induced emission (AIE) dyes have drawn increasing attention for their potential medical applications [64]. The dye molecules inside of the NP are protected from ROS in the surrounding environment and thus are very stable [65]. Unfortunately, most of the commonly used dyes such as fluorescein are highly emissive, but once inside the NPs, the fluorescence is quenched due to π–π interaction [65]. This aggregation-caused quenching (ACQ) effect determines the maximum amount of dye that can be loaded into each NP. Therefore, the quantum efficiency of the NPs cannot be increased by simply increasing the concentration of the dye [65]. The discovery of aggregation-induced emission (AIE) phenomenon has been vital in the area of fluorescence [65]. Different from the ACQ dyes, the AIE luminogens (AIEgens) are not luminescent when dissolved, but are turned “ON” when aggregates are formed [65]. In AIEgens, radiative decay is favored due to restriction of molecular motion in the aggregate state [65]. The non-planar conformations of the AIEgens prevent π–π interaction [65]. Restricted movement and non-planar conformation are vital for the strong emission of AIE dyes in the aggregate state [65]. AIEgens exhibit different emission colors spanning the visible region to the near-infrared [65]. There are two major methods for constructing luminescent polymeric nanoparticles (LPNs) based on AIE dyes: non-covalent methods and covalent methods [64]. The non-covalent methods for preparing LPNs involve embedding the AIE dye into a biocompatible amphiphilic polymer [64]. Unfortunately, the surface coating is easily separated, as well as the AIE dyes leaks out of the LPN restricting the use of non-covalent systems [64]. Alternatively, covalent strategies to prepare LPNs include reversible addition fragmentation chain transfer polymerization, emulsion polymerization, and anhydride ring-opening reaction [64]. Based on covalent techniques, AIE-active LPNs with uniform size, biocompatibility, water dispersibility, and intense luminescent emission have been synthesized and utilized for cell imaging [64]. Some typical AIE fluorogens include siloles, tetraphenylethylene (TPE), cyano-substituted diarylethlene, triphenylethylene, and distyrylanthracene [66]. Also, AIE molecules can be directly attached to a polymer/biomacromolecule, creating AIE dots [65]. For example, the isothiocyanate (ITC) group on (TPE-ITC) can react with the amino group on CS [65]. The fluorescence output of the obtained hybrid (TPE-CS) can be enhanced by simply increasing the TPE labeling ratio [65]. Also, a novel fluorescent sensor was constructed by He et al. [67]. That fluorescent sensor involved the hexaphenylsilole, which is a well-established AIE system that was doped into CS, and the resulting fluorescent film was used for demonstrating the selective detection of picric acid. The selectivity of the fluorescent CS film was attributed to the selective electrostatic interaction of the film with the picrate anion and screening effect of CS film on the interferents. In these films, the network of the CS film stabilized the fluorescent emission of the hexaphenylsilole by preventing further aggregation of silole aggregates [67].

#### 2.1.5. Metal Nanoclusters

Metal nanoclusters, usually ranging in size from several atoms to tens of atoms, have received enormous attention in the last decade [51]. The metal nanoclusters show special properties such as discrete electronic states, size-dependent fluorescence, and intrinsic magnetism [51]. Gold nanoclusters specifically exhibit excellent properties quite different from QDs and traditional organic dyes, such as low toxicity, good chemical and photo-physical stability, easy synthesis, and tunable fluorescence [51]. The bright NIR fluorescent AuNCs-BSA nanosystems produced by a simple one-pot green synthesis protocol has shown great potential in bioimaging and biochemical sensing [51]. However, AuNCs-BSA has been reported to be oxidatively decomposed by ROS and degraded by proteases or other enzymes in lysosomes [51]. Therefore, CS was chosen as a coating on the surface of the gold nanoclusters to improve the resistance to ROS and proteases [51]. AuNCs are sometimes fabricated onto solid platforms to form reusable sensors. In one of the cases, avoiding usage of the glass substrate, a free-standing film containing gold nanoclusters was prepared from CS polymer by the simple dip-coating process [61]. The AuNCs stabilized by glutathione were incorporated into CS film by chemical interaction of amino groups of CS with carboxyl groups of glutathione. In these films, the emission from AuNCs was quenched depending on the concentration of Cu^2+^ addition. Levels of Cu^2+^ were detected in the ppm range based on this luminescence quenching system. Also, the free-standing composite film was portable and more useful for practical applications with high stability [68]. In addition to AuNCs, AgNCs also show intense fluorescence in solution and are used in similar applications [69]. AgNCs have been prepared via several routes, such as chemical or photoreduction, etching, and micro-emulsion methods [69]. Selection of suitable stabilizing capping agents is the key issue for synthesizing AgNCs in order to avoid their aggregation [69]. Dendrimers were formally used for preparing AgNCs with high quantum yield. However, the Ag atoms formed large particles instead of clusters, which limited the scope of their applications [69]. Therefore, CS was used as a stabilizer with the desired properties of biodegradation and low-toxicity, and with the capability of controlling the sizes of the AgNCs obtained [69].

#### 2.1.6. Luminescent Organometallic Complexes

Luminescent metal complexes comprised of a transitional metal center or lanthanide metal centers have attracted intense interest in last few decades. Two of the biggest challenges for using luminescent complexes, especially for various biological applications, are their issues related to solubility and stability in aqueous media. Many works have shown the value of using polymers to overcome these issues. In many previous works, different polymers have been shown to enhance solubility, stability, and the photoluminescence properties of many organometallic complexes [46,70]. Polymers are selected based on chemistry and the desired application of the luminescent complex.

Accurate temperature measurement is a key to success in many chemical reactions and in many research fields. Temperature based luminescence changes are observed in many organometallic luminescent complexes for various reasons. Such complexes when fabricated as sensors can offer a solution for those applications in which traditional (mechanical, electrical, or IR-based) thermometers struggle. Ruthenium-based organometallic complexes are the most sought-after systems for sensing applications among the organometallic indicator dyes. Tsvirko et al. [71] have reported the development of a luminescent temperature sensor constructed by incorporating luminescent ruthenium (II)-tris (2,2′-bipyridyl) ([Ru(bpy)_3_]^2+^) into a CS polymer matrix. The sensor was shown to exhibit completely reversible and stable emission response and strong temperature sensitivity [71,72]. Takatoet al. [73] have shown the construction of an optical humidity sensor by understanding the effect of humidity on the photoluminescence (PL) of the Ru(bpy)_3_^2+^ system doped into CS. In this work, the authors have observed that electrostatic and hydrophobic interactions between the dye and the polymer played a significant role that determined the changes in PL vs. humidity. While understanding the role of the CS polymer, the authors have found that the PL quenching effect noticed in the polymer medium is due to O_2_ dissolved in the water phase of the polymer domain [73]. In the case of rhenium complexes, there is very limited literature on luminescent CS hybrids. Chakraborty et al. have used a luminescent rhenium carbonyl complex to graft onto a biocompatible carboxymethyl CS matrix to release CO, based on UV illumination to effectively eradicate human adenocarcinoma cells (HT-29) in a dose-dependent fashion. The system was shown to exhibit strong orange emission and release of CO upon exposure to low power UV illumination [74]. In the case of lanthanide-based systems, a CS-based hydrogel containing Europium (III) complex was prepared in the presence of poly(acrylic acid) using a regular *N*,*N*’-acrylamide crosslinking agent [68]. In the case of LaF_3_/Eu^3+^ luminescent nanoparticles, the coating of CS not only enhanced their biocompatibility and stability but also provided extra functional groups on the surface of these luminescent nanoparticles for further conjugation with biomolecules [68].

In one of our works, the amine-based functional groups on acidic CS have allowed for electrostatic interaction with a heavily sulfonated Au(I) molecular system (Figure 4). The Au(I) molecular system in aqueous media is weakly emissive. However, upon doping into a 0.5–1.0% *w*/*v* CS solution, the emission was enhanced by an order of magnitude. A clear ON-OFF behavior was noticed in the presence vs. absence of the CS polymer. Depending on the concentrations of both the Au(I) molecular system and the CS polymer, formation of phosphorescent nanoparticles was also noticed. Compared to fluorescent systems, phosphorescent systems are advantageous [46,70] due to their long emission lifetime (micro to millisecond), which is favored in bioimaging applications to overcome autofluorescence of biological agents or cells. Such phosphorescent systems are also highly favored in sensing applications, because of the stable lifetime signal that is insensitive to experimental conditions compared to the emission intensity signal. In both imaging and sensing applications, long phosphorescent lifetime is highly preferred compared to a short fluorescence emission lifetime. In a very recent work, our group was able to take advantage of CS solubility in acidic aqueous media and was able to use CS to stabilize a Au(I) trinuclear molecular system. For the first time, our group has demonstrated the formation of a phosphorescent Au(I) cyclic trimer complex, synthesized and stabilized in CS polymer. When synthesized in aqueous media, the complex was only stable for a few hours. However, when synthesized in CS polymer, this complex was stable for months. Formation of such Au(I)-based trimers based on different ligands and their interesting photophysical properties is well documented, although the formation of such trimers in aqueous media with retention of their photoluminescent properties has not been previously demonstrated. When stabilized in CS media, the complex exhibited complete retention of environmentally sensitive photophysical properties such as retaining its ability to sense heavy metal ions in solution, as well as a changing pH. Based on the above-cited works, we strongly believe that these results highlight the potential for CS polymer usage for stabilizing photoluminescent molecular systems that are often unstable in aqueous media. Such systems stabilized in polymer-aqueous media can find extensive usage in optical sensing and imaging fields. See Table 1 for summary of different CS polymer based fluorescent/luminescent composites.

### 2.2. Plasmonic Metal Nanoparticle–Chitosan Systems

Metal nanoparticles are known to have unique properties based on their size and shape [78,79]. In case of metals, there is no separation between the conduction and valence bands. In metals like gold, silver, copper, and platinum, the decrease in the size of the metal, below the electron mean-free-path, results in intense absorption or scattering due to coherent oscillation of the free surface electrons, known as plasmons in the conduction band. Plasmons can be described as the oscillation of free electron density against the fixed positive metalcore. Surface plasmon resonance (SPR) is what causes these metal nanoparticles to exhibit intense absorption and/or characteristic colors. SPA is also referred to as localized surface plasmon resonance (LSPR), since it is localized at the surface of the metal. This LSPR characteristic is exhibited by noble metal nanoparticles and has renewed the interest of physicists, chemists, materials scientists, and biologists in surface plasmonic nanoparticles. This intense absorption or LSPR is highly sensitive to the size and shape of the nanoparticles. The absorption is also sensitive to the pH, refractive index, solvent, and dielectric constant of the surrounding medium of the nanoparticles. The tunable properties (including size and shape) and environmental sensitivity of these plasmonic nanoparticles are being adapted to different applications including sensing, imaging, delivery, diagnostics, and therapy [78,79,80]. The plethora of applications for plasmonic nanoparticles has initiated the development of numerous approaches for synthesizing tailor-made sizes of stable, SPR-tunable nanoparticles in different mediums of interest. As it is very difficult to extensively cover all plasmonic nanoparticles, the focus of this review will be on gold and silver nanoparticles.

Given, the unusual optical properties of plasmonic nanoparticles, the synthesis of these NPs is considered to be a major research area in nanotechnology. Michael Faraday’s preparation of gold sols in 1857 initiated the work on metal nanoparticles [78]. In this work, the red-colored colloidal gold solution was prepared by reduction of an aqueous solution of chloroaurate (AuCl_4_) with phosphorus in carbon disulfide (CS_2_) as a reducing agent. Following this invention, many methods for the preparation of gold colloids or AuNPs were reported over many years. Turkevich and Brust et al. [78] have separately refined and explained the process of nucleation, growth, and agglomeration of gold sols. Turkevich’s reduction of the Au(III) compound HAuCl_4_, either in the presence of NaBH_4_ or hot sodium citrate solution, is still the most popular method for making AuNPs [78]. The formation of these noble metal nanoparticles, especially from gold and silver, involves two major steps. The first is the reduction of metal salts, such as a chloroauric acid (HAuCl_4_) or silver nitrate (AgNO_3_), in the presence of a reducing agent, such as sodium borohydride (NaBH_4_), ascorbic acid, or citric acid. These reducing agents reduce the Au(III) and Ag(I) salts to Au(0) and Ag(0). Some of these stabilizers and/or reducing agents used for making spherical nanoparticles are less harsh compared to the strong surfactants and reducing agents that are involved in making size- and shape-controlled nanoparticles. Some of these chemical stabilizers like cetyltrimethylammonium bromide have been proven on multiple occasions to be cytotoxic to different cell lines and fish [81,82,83]. In the second major step of synthesis, the zero-oxidation state metal is nucleated and stabilized to form nanoparticles, during which the role of the stabilizing agent becomes very critical for keeping the nanoparticles stable in solution. Due to high surface energy, the nanoparticles tend to attract and agglomerate in solution at short interparticle distances, resulting in a loss of the characteristic optical properties. Stabilization is generally achieved sterically or electrostatically using special molecules or solvents. Both in aqueous and organic medium, stabilizing molecules act as strong adsorbents and prevent agglomeration. The use of metallic nanoparticles as structural and functional units for all the applications depends on the functionality of these stabilizing units [84]. Among different ligands, thiol-based ligands are highly recommended for their strong affinity to bind with gold due to the soft character of both Au and S. Other sulfur based ligands like xanthates and disulfides have been used to stabilize AuNPs. Non-sulfur ligands like phosphines, phosphine oxides, amines, and carboxylate ligands are also known to stabilize gold and silver nanoparticles [78,84]. In many cases, it is observed that a single polymer or a ligand can act as both reducing and stabilizing agent depending on the chemistry and affinity of the ligand or the polymer for the metal surface. Despite promising applications and growing environmental concerns (especially in the case of AgNPs), it was found that less than 24% of reported methods completely rely on techniques that are environmentally friendly for producing particles smaller than 20 nm [85]. In addition, the stability of these nanoparticles in the environment or biological media is also under discussion. With an ever-increasing focus on the biological and environmental safety of these nanomaterials, producing these nanomaterials following “green” chemistry principles have gained significant attention [86,87]. Some of these “green” principles include (a) synthesis in an aqueous solution; (b) elimination of toxic impurities from reactants and products; and (c) containment of reactive chemical groups to simplify subsequent attachment to biomolecules. Finally, a “green” method for nanomaterial preparation should pass an evaluation in four primary areas: the solvent, the precursor, the stabilizer, and the reducing agent. 

Investigations on the surface modification of metallic nanoparticles with biological polymers showed highly desirable results in terms of decreased cytotoxicity and differential targeting of cell lines [88]. To expand and potentially leverage off of these results, different techniques have been employed to overcome toxicity issues and environmental concerns of these metallic nanoparticles. Many researchers have reported the synthesis of these plasmonic nanoparticles using non-chemical reducing agents like radiation, sonication, and heat. These non-chemical energy sources allow for reduction of metal salts [78,79,84,89] in the absence of a chemical reducing agent. However, selecting a biological and physiologically compatible stabilizing medium is still challenging for making size- and shape-tunable, optically active NPs. Understanding these limitations, some research groups have started employing various kinds of biological polymers, plant-animal extracts, and other benign materials extracted from biological sources (like DNA, oligonucleotides, and amino acids) for stabilizing both silver and gold nanoparticles [78,79,81,82,83]. One of the methods heavily investigated to eliminate harsh chemicals so as to fit within the “green” chemistry principles is the use of plant extracts. There have been multiple reviews outlining the use of plant extracts for stabilizing metallic nanoparticles. In 2014, Ikram et al. [90] explained the role of plant-based biological molecules for making AgNPs. The plant extracts are envisioned to undergo highly controlled assembly that makes them suitable agents for stabilizing AgNPs or any metallic nanoparticles. In this review, the authors addressed the issue of toxicity and biological risks from general chemical and stabilizing agents. They explored the huge plant diversity that has been employed for making AgNPs of different sizes and shapes using single step preparation techniques. The review lists more than 20 types of plant extracts that have been used for making different size and shaped silver nanoparticles, terming all these different methods as “green” synthesis methods [90]. In 2015, the same group summarized the role of different plant extracts for stabilizing AuNPs of different sizes and shapes. In these two reviews, the authors have very clearly tried to overcome limitations of existing chemical synthesis methods for making environmental friendly and biocompatible gold and silver nanoparticles of different sizes and shapes [91]. In 2014, Pasca et al. have shown the formation of spherical and polygonal shape gold nanoparticles using different plant extracts (from Angelica, Hypericum, and Hamamelis), naming their synthesis approach as biogenic [92]. In this context, the usage of leaf or plant extracts fits within the “green” chemistry terminology, but many believe that the approach suffers from inconsistency due to significant variations in the chemical composition of the extracts. Also, on many occasions the method fails to explain the precise mechanism of the formation of nanoparticles, which is likely due to the complicated biomolecular mixture associated within these extracts [90,93,94,95]. Additionally, most of the synthesis methods involving plant extracts resulted in the formation of a mixture of spherical and anisotropic particles. For applications requiring the presence of a single size and shape monodispersed nanoparticles, methods using plant extracts are not considered to be a viable option. Polymer chemists are continuously trying to address this issues of producing monodispersed, single size, and shape plasmonic nanoparticles by employing biocompatible polymers (such as polyethylene glycol (PEG), Mannan, and CS) that are expected to overcome these limitations, yet fit within “green” chemistry principles.

#### 2.2.1. Chitosan Containing Silver Nanoparticles

Among plasmonic nanoparticles, silver nanoparticles are the most widely-commercially used nanosystems. Recent reviews on prospective applications and environmental concerns of AgNPs have summarized their broad usage ranging from consumer products to disinfecting medical devices and home appliances (see Table 2) [105]. AgNPs are also widely studied for their superior catalytic activity, surface-enhanced Raman spectroscopy (SERS), and metal-enhanced fluorescence (MEF) applications. SERS and MEF are considered powerful tools for high-throughput screening, immunoassays, and macromolecular detection dictated by the size and SPR of AgNPs [103,104]. In medical fields, the emergence of antibiotic-resistant bacteria has attracted the strong attention of researchers in the field of AgNPs, because they possess broad anti-inflammatory, antifungal, antimicrobial, and wound healing properties. Due to the fact that AgNPs can interact with more than one type of cell constituent, AgNPs has gained popularity in antibiotics research [106]. In some cases, AgNPs in combination with antibiotics have been shown to exhibit a synergetic antimicrobial effect on bacteria and pathogens. Work by Navi et al. has shown an average of a 2.8-fold increase in antibacterial activity using a combination of antibiotics with AgNPs. In this work, the group synthesized the AgNPs stabilized from the culture filtrate of the *Aspergillus flavus fungi* [107]. The ability of AgNPs to act as antitumor agents is also well demonstrated. Sriram et al. have successfully demonstrated antitumor properties of biologically synthesized AgNPs in Dalton’s lymphoma ascites (DLA) cell lines, both in vitro and in vivo [108]. Applications of these AgNPs are numerous and widespread (see Table 2).

**Figure 5:**
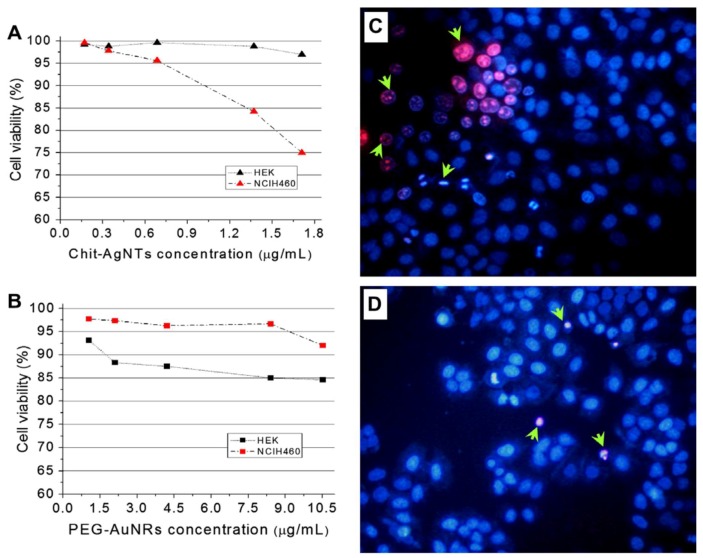
Demonstrates stabilizing feature of CS polymer during formation of CS capped silver nanotriangles. The CS-AgNTs are shown to exhibit better biocompatibility compared to PEG stabilized AuNRs. The figure shows (**A**,**B**) the cytotoxicity profiles of CS-stabilized AgNTs and PEG-AuNRs respectively towards HEK (black symbols) and NCI-H460 (red symbols) cells. NCI-H460 cells double-stained with Hoechst-viability and Propidium Iodide-mortality indicators the presence of (**C**) CS-AgNTs and (**D**) PEG-AuNRs. The arrows indicate condensed and fragmented nuclei typical of apoptotic cells. Reprinted with permission from *Cancer Lett.* **2011**, *311*, 131–140, Copyright 2011, Elsevier Ireland Ltd. [115].

Due to the antibacterial activity of CS, coating the AgNPs surface with CS has been reported to enhance the antimicrobial activity of these nanoparticles. A recent survey of SciFinder Scholar (30 March 2018) conducted herein for the research topic “Chitosan Silver Nanoparticles” has resulted in 918 articles. These 918 references involved applications ranging from dressing scaffolds, nanofibers, water treatment, drug delivery, and even catalysis, although most of these articles were dominated by antibacterial and wound dressing applications of AgNPs. There were also research articles dealing with composite materials for dual function, such as AgNPs containing graphene oxide for both antibacterial and catalytic activity. Summarizing each and every article on AgNPs is beyond the scope of this article. Some authors have selectively summarized the works dealing with CS polymer. In one of the early papers on this work, Huang et al. in 2004 have shown the formation of both AuNPs and AgNPs within polysaccharides. The polymers are shown to act as both reducing and stabilizing agents. They obtained positively charged AuNPs using CS as a stabilizer and negatively charged AgNPs using heparin as a stabilizer. In this work, the authors also explained the significance of stabilizing the gold and silver nanoparticles within these biologically benign reagents [14]. Citing some previous works on employing fungus, glucose, and starch, the authors claimed to stabilize isotropic AuNPs within CS alone in the complete absence of any other reducing agents [14]. In 2011, the “green” synthesis of silver nanocomposites stabilized in CS and PEG was reported by Ahmad group. In this work, the CS was used as solid support and polymeric stabilizer. The authors explained the significance of avoiding usage of reducing agents like NaBH_4_ for developing “green” synthesis methods. Formation of different size AgNPs at different stirring times at 60 °C was evaluated in this paper and found that shorter stirring time resulted in smaller AgNPs [15]. In 2011, while reporting a synthesis method for making silver nanoparticles within CS, Honary et al. have observed that the molar mass of CS has a noticeable effect on the size of AgNPs. Also, their study reported achieving higher antibacterial activity against *Staphylococcus aureus* with smaller sized particles, due to the enhanced surface area. In this report, authors employed NaBH_4_ to ensure complete reduction of silver ions [13]. In 2016, Ahmed et al. reported the potential usage of CS, CS derivate, and CS metal nanoparticles in pharmaceutical drug delivery and have reported the significance of stabilizing the AgNPs in CS. In this review, the enhanced antibacterial activity and concerns related to human and environmental safety from the usage of these metal nanoparticles are discussed [109]. In a recent article published in 2017, Ryan et al. have investigated the addition of metal nanoparticles to cross-linked CS composites of siloxane-CS to produce materials with enhanced antibacterial properties [110]. In some of these works cited above, CS is shown to exhibit a triple role as a solvent, stabilizer, and reducing medium. Some researchers have extensively demonstrated the ability of CS to reduce and stabilize different sized AgNPs within CS medium in the absence of any other reducing or stabilizing media. For the development of novel wound management materials, several challenges related to adhesion to wound surface, absorbing exudates, and enhancing bactericidal effectiveness need to be addressed. Only a few current biomaterials are known to address all of these characteristic properties. In 2016, Rinehart et al. developed a CS/PVA ((poly)vinyl alcohol)-based hydrogel composite for responsive wound management. In this work, the authors have optimized the CS/PVA ratio by deposition method for incorporating Ag^+^ and pH of the composite. This optimization has helped achieve the gradual release of Ag^+^, which increases the longevity of the wound dressing substrate [111]. In a similar work by Pei et al., the impact of AgNPs in wound dressing application was investigated using AgNPs-loaded silk fibroin/carboxymethyl CS composite sponge. The introduction of AgNPs into biological dressings was demonstrated to be a beneficial method to prevent wound infection and promote wound healing [112]. Hien et al. investigated the influence of CS polymer for binding the AgNPs to cotton fibers [113]. The spherical AgNPs with sizes less than 10 nm were synthesized by Co-60 ray irradiation in CS media and later incorporated onto cotton fabric. The results from antibacterial activity against *S. aureus* showed that AgNPs incorporated onto cotton fabric exhibited the highest antibacterial activity [113]. Another very interesting subsection of plasmonic nanoparticle research is plasmonic nanoparticles with anisotropic shapes. These are highly sought-after for biomedical applications due to their ability to absorb and/or scatter light strongly in the NIR region (ca. 800–1350 nm). Other than direct biomedical applications (e.g., photothermal therapy, photoacoustic imaging, image-guided therapy, and drug delivery) the ability of these anisotropic plasmonic nanostructures to extend plasmonic resonance beyond visible wavelengths was found to be extremely significant for technologies related to high-efficiency solar cells, infrared photodetectors, heat-absorbing optical coatings, and sensors. For chemical sensing and imaging, nanoparticles that absorb/scatter in the NIR region are believed to possess distinct advantages compared to nanostructures that absorb in the visible region [114]. Very limited progress has been achieved for synthesizing these most challenging and intriguing, shape- and size-specific silver nanoparticles within a CS medium [114]. In general, such large and anisotropic plasmonic nanoparticles are synthesized using toxic surfactants like CTAB (cetyltrimethylammonium bromide), poly(vinyl pyrrolidone) (PVP) polymer, or the bis(p-sulfonatophenyl)phenylphosphine dihydrate dipotassium (BSPP) salt. One of these very common stabilizing media, CTAB is a well-established cytotoxic surfactant. Replacement of such chemicals by a biocompatible polymer like CS would be a great advancement, and warrants further usage of such attractive NIR sensitive plasmonic AgNPs for various biomedical applications. Based on our literature search, we found only a few works for stabilizing NIR absorbing AgNPs within a CS system. Work by Potara et al. [115] described the formation of large, anisotropic-shaped AgNPs within CS polymer by following seed-mediated chemical reduction protocol, which involves sodium borohydride, trisodium citrate, and ascorbic acid. Using the thus-formed anisotropic AgNPs, the authors have demonstrated the SERS efficiency of the biocompatible films, which is assessed by Raman imaging of adenine. The Raman intensities were greatly enhanced due to the presence of CS. This study demonstrates the very effective application of CS-based plasmonic films as substrates for SERS detection of non-resonant analytes at the single-molecule level. Followed by Potara’s work, Boca et al. [116] demonstrated the significance of Potara’s anisotropic particles by employing selectively λ ≤ 750 nm-absorbing AgNPs as photothermal transducers for cancer cell therapy. In this work, the CS-coated silver nanotriangles (AgNTs) with strong resonances in the NIR region operated as photothermal agents against a line of human lung cancer cells (NCI-H460). In the photothermal therapy experiment, the nanoparticles were excited at 800 nm and found that cell death was higher in the presence of CS-coated AgNTs compared to PEG-coated gold nanorods (Figure 5). The CS-coated nanotriangles were also found to exhibit good biocompatibility to healthy human embryonic cells (HEK). This work illustrates the impact and significance of CS-stabilized, NIR-absorbing AgNPs for cancer research. Additionally, the only other work we found on usage of CS for stabilizing NIR AgNPs is the recent work from our group. In this work, we employed CS polymer to synthesize strong NIR tunable anisotropic AgNPs by following a photochemical synthesis method. In this work, we have used CS both as a synergetic reducing agent and stabilizing agent for making “worm”-shaped anisotropic AgNPs that absorb very effectively within the NIR region. We found that the silver nanoworms retained their NIR-absorbing features even at physiological pH and at a constant ionic strength. The silver nanoworms exhibited highest growth inhibition compared to spherical nanosilver and molecular silver forms on gram-negative plant pathogenic bacteria, *Pseudomonas syringae* pv. *maculicola* ES4326, and *P. syringae* pv. tomato DC3000. The results from this work suggested that nanoworms favor the adhesion to (curved) rod-shaped, gram-negative bacteria, resulting in higher inhibition than isotropic AgNPs (smaller spheres), sulfa antibiotics (silver sulfadiazine), and silver ions (AgNO_3_). Results from limited works on using CS for stabilizing NIR or anisotropic AgNPs have validated the extensive potential of CS polymer as a perfect stabilizing agent for making highly intriguing NIR absorbing AgNPs. We strongly believe that there is still abundant scope, and opportunities are still available in this specific area of research.

#### 2.2.2. Chitosan Containing Gold Nanoparticles 

In view of tremendous applications of AuNPs (Table 2), it is highly desirable to identify new “green” synthesis methods to provide much better biological compatibility between AuNPs and bimolecular moieties. Therefore, a lot of biologically friendly approaches are constantly being developed for both the synthesis and surface modification of AuNPs. For example, the use of proteins, microorganisms, or plant extracts to produce different types of biologically friendly hybrid gold nanoparticles has become an area of research with great potential. Additionally, some biodegradable polymers, either natural or synthetic, have been investigated with the aim of generating, stabilizing, and templating AuNPs. Among natural polymers, CS is attracting attention due to its unique biological and physicochemical characteristics. Huang et al. found that CS not only acts as a protecting agent but as a reducing agent as well, helping reduction of tetrachlorate gold salt to gold nanoparticles in the complete absence of any special reducing agent. The formation of AuNPs was reported within CS in the presence and absence of TPP [117]. The paper reported that the shape and size distribution of AuNPs can be altered by changing the molecular weight and concentration of CS. Moreover, the paper also reports the effect of “gelation” resulting from TPP addition to CS. This gelation helps tune both size and shape of the AuNPs. Additionally, the authors have found that by varying the concentration and molecular weight of CS, some polygonal shape AuNPs exhibiting two SPR bands are easily developed [117]. This work very clearly established reducing behavior of CS for preparation of AuNPs. The mechanism of gold ion reduction in CS solution and the effect of temperature on the formation of CS-embedded AuNPs was also studied [117]. On the other hand, there are many investigations in the literature dealing with understanding the ability of CS polymer to stabilize different sized AuNPs, exclusively for surface-enhanced Raman scattering (SERS) application. A work by Potora et al. investigated the formation of CS-embedded AuNPs at different temperatures and discovered that temperature plays a crucial role in formation and stabilization of AuNPs [118]. Additionally, stability and aging of AuNPs solution, functionalized with CS polymer in relation to pharmaceutical usage, is illustrated by Raoul et al. In this work, the group stabilized different sized AuNPs within CS and evaluated their physiochemical properties and stability at room temperature in aqueous solution by following SPR absorbance and zeta potential values through a period of 24 months. They determined that AuNPs coated with CS of medium molecular weight and medium concentration demonstrated better colloidal stability [119]. In a work by Le et al., the usage of CS-stabilized AuNPs for electrocatalytic oxidation of uric acid was demonstrated. In this work, the Le group successfully prepared AuNPs with water-soluble CS, and later a newly modified electrode was fabricated by self-assembling AuNPs to the surface of the l-cysteine-modified glassy carbon electrode [120]. In another work, Sugunan et al. reported a novel strategy for sensing ions of heavy metals using AuNPs capped with CS. The negatively charged AuNPs were stabilized by the polycationic nature of CS through electrostatic interactions. The role of CS was to provide steric hindrance and to functionalize the nanoparticles for use as sensors. The combination of the chelating property of CS and optical properties of AuNPs has been synergistically applied to detect low concentrations of heavy metals ions like zinc and copper in solution [121]. In another sensing work by the Curulli group, gold-CS nanocomposites were successfully applied as selective electrochemical sensors for the determination of the antioxidant caffeic acid, which has attracted much attention due to its benefits on human health. In this paper, taking advantage of the sensing mechanism of this nanocomposite, an analytical method for determination of polyphenol index in wines was proposed [122]. In a very interesting work, the effect of CS-stabilized plasmonic nanoparticles is investigated to understand penetration and uptake of therapeutic agents such as insulin, across the mucosal membrane [109]. Very interestingly, the work showed that insulin-loaded CS gold nanoparticles were stable for more than 6 months and significantly lowered the blood glucose level in diabetic rats following oral and nasal administration [109]. In another exciting study, Yong Hu et al. have discussed the formation of CS-PAA-Au hybrid nanospheres via the one-pot route in aqueous media. These hollow spheres were shown to act as intracellular and intranuclear drug delivery agents. Additionally, they were shown to act as contrast agents in cancer cells lighting up the nucleus and as anticancer drugs at the same time. This multifunctionality is highly promising in cancer research, because the hollow spheres are shown to possess plenty of functional groups with the ability for surface functionalization [123]. Similar to AgNPs, work on the stabilization of NIR-sensitive or anisotropic-shaped AuNPs within CS are very limited. Nandanan et al. have demonstrated the formation of gold nanorods (AuNRs) using functionalized CS. The same group, while explaining the formation of functionalized AuNRs, has listed the challenges related to the development of functionalized AuNRs. Formation and surface functionalization of AuNRs with CS oligosaccharides is reported. The CS oligosaccharides containing multiple amine and thiol groups are found to help provide multiple binding sites for robust coating and protection against aggregation [124]. We also found that there are multiple reviews on polymer-stabilized AuNPs in literature. In most of these reviews, the focus was on explaining the stabilization of AuNPs with both biopolymers and also with artificially synthesized polymers. Much effort was also spent on explaining the stabilization of AuNPs using biological materials like nucleotides and DNA. In a review published by Ofir et al. in 2008, authors tried to summarize the gold nanoparticle–polymer composites by detailing the synthesis, optoelectronic applications, and sensing devices made therein using AuNPs-polymer composites. In this extended review, the authors focused on a variety of polymers including DNA, proteins, virus, ssDNA, dendrimers, self-assembly systems (e.g., poly(ethylene imine), block copolymers (e.g., polystyrene-b-(methylacrylate)), polymethylmethacrylte, and polystyrene matrices. They explained extensively the self-assembly and various other approaches of formation of polymer-AuNPs composites and their application in sensors and memory devices. However, there was no mention of CS in that review, since we believe that CS-based AuNPs were still in the research and developmental stages at that point [125]. In 2010, a review by Uehara summarized the sensing properties of gold nanoparticles conjugated with functional polymers. In this review, the author differentiated the conjugation of AuNPs with polymers into two types: one based on biopolymers and the other based on artificial polymers. The fluorescent detection and sensing properties of AuNPs are highlighted in this review [126]. In a review in 2007 by Shan et al., the advances in polymer-stabilized AuNPs from 2004 to 2007 were listed, and their increasing and attractive applications were summarized. In this review, the authors described the stabilization of AuNPs extensively with different types of ligands containing hydroxyl, carboxylic acids, and glycidyl groups. Different approaches explaining anchoring of AuNPs to these different ligands were explained in detail but were restricted to only one citation highlighting the advantage of CS as a benign polymer for stabilization of AuNPs [89]. In 2007, Bajpai et al. published a review in the Journal of Nanoscience and Nanotechnology describing the synthesis and stabilization of silver and gold nanostructures in polymer media. In this review, the authors explained the significance of polymer-stabilized gold and silver nanoparticles. They discussed a variety of molecules including inorganic salts, organic compounds, organic solvents, and biological systems as stabilizing agents. Again, there was only a single citation explaining the role of CS-stabilized AuNPs for heavy metal sensing described in that review [127]. From the above highlights, we would like to point out that though there are numerous, extensive reviews of AuNPs stabilized in different polymers, a review of stabilized AuNPs exclusive to CS and its derivatives is uncommon. To the best of our research, we found more than 150 references in SciFinder Scholar using the title search “Chitosan Stabilized Gold Nanoparticles”, but we strongly believe that there are only a few specific direct works including CS and AuNPs. Search results would likely result in a much larger number of citations if CS derivatives containing multi-metal- or metal-oxide AuNP hybrids were considered. Understanding that all of the different types of metal nanoparticles containing CS cannot be covered in this review, some very limited works on other metal nanoparticles are also included. Some of these works are centered on taking advantage of the synergetic effects of both CS and metal nanoparticles. Salehizadeh et al. [108] mentioned the formation of Fe_3_O_4_–gold–CS nanostructures. These hybrid nanostructures containing both gold and iron oxide nanoparticles were prepared using a coprecipitation method. The prepared nanoparticles using this method were also found to be highly attractive for various biomedical applications [109]. In a different work, copper-loaded CS nanoparticles were prepared by ionotropic gelation. The copper-containing CS nanoparticles exhibited marked growth inhibition of a wide range of microorganisms, such as *S. aureus*, *Salmonella typhimurium*, *Salmonella choleraesuis*, and *Escherichia coli*, in which the minimum inhibitory concentration was less than 0.25 μg/mL [108]. There are some rare works on employing “green” synthesis to prepare copper nanoparticles. These involve reduction of copper in an aqueous solution of CS and an organic acid, such as ascorbic acid, which prevents the formation of copper oxides [109]. The research area of SPR is a very extensive field, and there are many other nanosystems that exhibit interesting optical properties due to SPR. Copper nanoparticles, gold nanoshells, silver nanoshells, gold-silver nanoshells, platinum, and palladium nanorods [128] are some examples. For more details, see Table 3 for a list of different synthesis methods and applications of gold and silver nanoparticle composites containing CS polymer. 

### 2.3. Some Selective Applications of Chitosan-Based Photonic Systems

Many different types of photonic applications are in the literature utilizing these optically active CS systems. Therefore, we have selected a few of the most significant of these applications and have discussed them in detail below.

#### 2.3.1. Bioimaging and Cancer Research

Though there are multiple applications of luminescent CS hybrids, we have selected the most prevalent works. Although great progress has been achieved relative to the diagnosis and therapy for various cancers in last decades, cancer remains one of the leading causes of death [75]. Patients treated with conventional chemotherapy commonly suffer from severe side effects [75]. One of the goals of chemotherapy is to develop a drug delivery system that can intelligently trigger the drug release targeted at the cancer cells, to reduce the drug’s side effects on the patient and improve the overall therapeutic efficacy [75]. Using photothermal therapy (PTT) light-absorbing agents for converting NIR light into heat under laser irradiation offers a unique potential alternate approach. Specifically, extensive attention to cancer treatment is focusing on these PTT light absorbing agents, which could convert NIR light into heat such that a sufficient temperature could be reached to kill cancer cells [129]. To have a safe and effective treatment, it is crucial that the location and size of tumors be monitored by imaging before, during, and after therapy [129]. An ideal cancer PTT should kill the treated tumors, induce a systemic antitumor immunity, control metastatic tumors, and promote tumor resistance [130]. Indocyanine green (ICG) is a NIR dye that has been approved by the United States Federal Drug Administration (FDA) for clinical imaging agents due to its advantageous photochemical, photobiological, and pharmacokinetic properties [76,129]. ICG absorbs at approximately 780 nm and emits around 800 nm, making it highly suitable for bio-imaging applications with high signal-to-background ratio [76]. Moreover, it can convert the absorbed light energy to produce heat for PTT treatment [76]. However, the use of ICG for imaging and PTT is limited, because ICG is photo-degraded in aqueous solution [76] and unstable at high temperature, resulting in loss of absorption and fluorescence. All of these disadvantages restrict its use in PTT [76]. To overcome these disadvantages, effort has been made to improve ICG’s photo and thermal-stability, pharmacokinetics, and biodistribution in tumor tissue [76]. Recently, a CS-based, ICG-containing nanostructure for effective molecular tumor imaging has been developed [76]. Song et al. synthesized CS-polyethylene glycol (PEG)-ICG nanoparticles by adding ICG to a CS-PEG aqueous solution, and the nanoparticles were formed through a self-assembly method via electrostatic interaction [76]. The resulting CS-PEG-ICG nanoparticles exhibited improved photo- and thermal-stability (compared to ICG alone), good biocompatibility, and low toxicity [76]. When irradiated with a laser, the tumor cells containing the CS-PEG-ICG nanoparticles showed only 15% cell viability in vitro photothermal toxicity. Also, the CS-PEG nanocarriers altered the biodistribution and prolonged the retention time of ICG in the mice after intravenous injection [76].

Carbon Dots (CDs) have also been studied for targeted release of cancer drugs. CDs with unique optical properties have been incorporated into different nanomaterials for applications as higher quality membranes, catalysts, drug carriers, MRI contrast agents, and nanodevices [75]. When these optically active nanoparticles are embedded into materials such as CS, the resultant hybrid material can be applied in the sensing field [75]. As mentioned earlier, compared with other optically active nanoparticles such as QDs, CDs are less toxic and cheaper [75]. Also, CDs can be used for PTT and NIR fluorescence imaging due to their NIR absorption and emission [75]. The crosslinking of CS chains in the nanoparticles is able to immobilize small CDs complexed in the CS networks [75]. The resultant CS-CD hybrid nanogels (CCHNs) have colloidal stability, high loading capacity for doxorubicin, bright and stable fluorescence ranging from the UV to the NIR, efficient NIR photothermal conversion, and drug release in response to both NIR light and pH change [75]. The results from tests on different tissues of an animal model indicate that the CCHNs are nontoxic [75]. The doxorubicin-loaded CCHNs are able to permeate into the implanted tumor on mice and inhibit tumor growth [75]. After CCHN treatment, additional photothermal treatments from NIR irradiation can further inhibit tumor growth [75].

#### 2.3.2. Sensing

**Figure 6:**
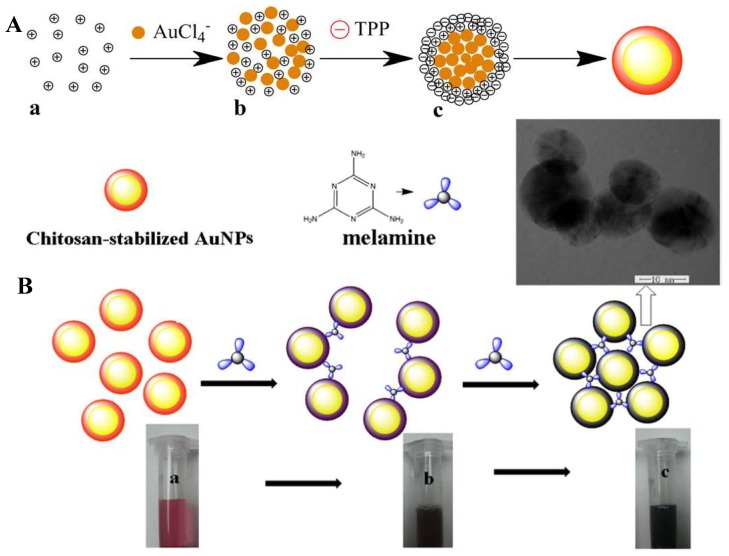
Demonstrates the sensing action of CS stabilized AuNPs. Schematics show the formation of CS stabilized AuNPs. (**A**) Schematic representation of the formation of chitosan-stabilized AuNPs where (**a**) represents the polycationic form of CS, (**b**) shows the formation of ion pairs with AuCl_4_^-^ and (**c**) shows the CS stabilized AuNPs. The red circle indicates the tripolyphosphate (TPP); (**B**) Schematic representation of colorimetric mechanism for melamine detection. The insert is photographs of solution of tubes (**a**) CS stabilized AuNPs, (**b**) CS stabilized AuNPs with melamine, (**c**) CS stabilized AuNPs with melamine and TEM image of chitosan-stabilized AuNPs with melamine. The melamine detection is indicated by the color change of AuNPs. Reprinted with permission from *Food Control*. **2012**, *32*, 35–41, Copyright 2012, Elsevier [137].

**Figure 7:**
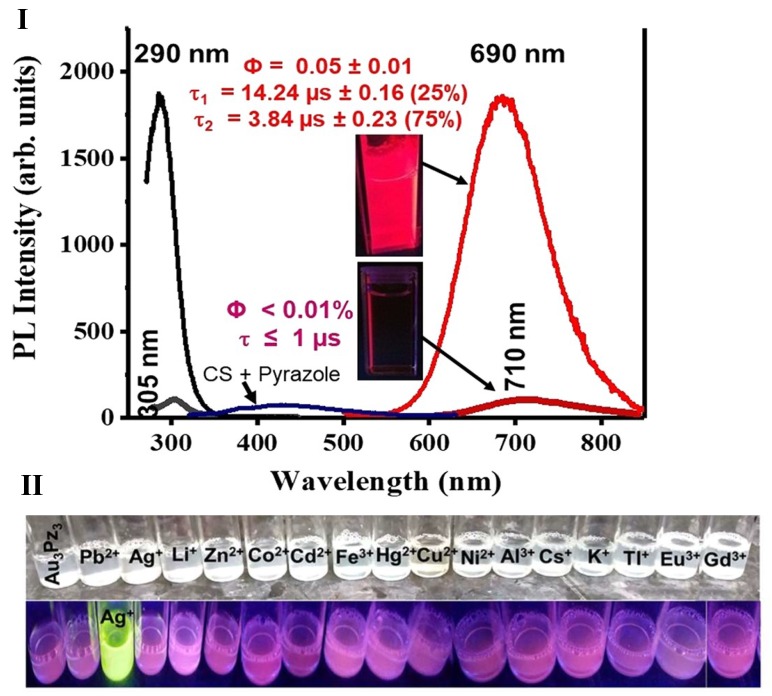

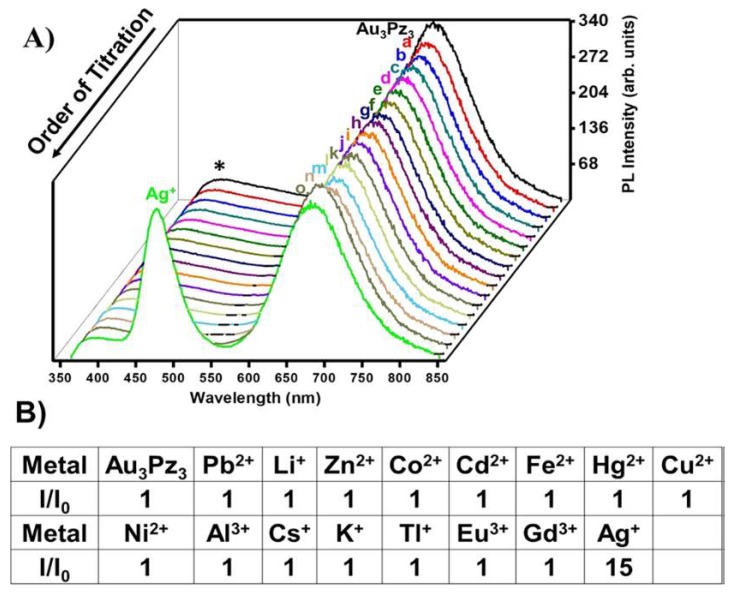
Demonstrates stabilizing feature of CS polymer. Formation of phosphorescent Au(I)-based molecular system and its heavy metal sensing application. In figure **I**, the difference in photoluminescence spectra of Au(I) molecular system stabilized in aqueous and chitosan media is shown. In figure **II**, the silver sensing ability of Au(I) molecular system stabilized in CS polymer is demonstrated from fluorescent images. (**A**) Changes in photoluminescence spectra of Au(I) based molecular system in the presence of various metals is shown; (**B**) Shows I/I_0_ values of various metals, derived from photoluminescence spectra. The “*” indicates weak emission from impurities in chitosan. Reprinted with permission from *Anal. Chem*. **2018**, *90*, 4999–5006, Copyright 2018, American Chemical Society [69].

Fluorescent or calorimetric dyes that undergo a change in color or fluorescent properties upon interaction with chemical species can be defined as an indicator or probe. In optical sensing, where light is used as a source for acquiring information, the indicators or probes will help convert the concentration of the analyte into a measurable optical signal. Indicators or probes transduce the signal for qualitative and quantitative analysis of the analytes. Upon interaction with the target analyte/species, the optical indicator molecules or nanomaterials will undergo a change in color or fluorescence. These changes are recorded using a suitable spectrophotometer [131]. This principle is applied to various platforms, such as very common fiber optic sensors. Whatever the platform, a polymer support to load the indicator is an integral part of the sensor. There are numerous polymers that are employed as polymer supports depending on applications and sensory criteria. On most occasions, an optically inert polymer is selected for loading the indicator dye, so there is no optical interference from the polymer support. Such color change of fluorescent indicators is heavily employed in pH sensing, heavy metal sensing, sensing of industrial and toxic gases (e.g., oxygen and CO_2_), temperature, and biologically important elements like Ca^2+^ [131]. Recent advances in sensing have allowed for visualizing the non-fluorescent moieties by tagging a fluorescent dye, a required development for sensing and imaging of optically inactive systems [132]. The hydrophilicity and non-antigenic nature of the CS biopolymer, which has low toxicity towards mammalian cell lines, offers great potential as a supporting media or stabilizing media for fluorescent systems. Such systems are shown to exhibit undisputed value in sensing and imaging applications [8,133]. Very recently, Baranwal et al. have summarized many sensing applications using CS polymer. The group explained the significance and impact of CS polymer for biosensing, highlighting its potential versus other biological polymers like poly-lysine, poly-glutamate, and alginic acid. The very desirable properties of CS for bio-sensing were summarized. Many optically active materials including AuNPs and AgNPs have been fabricated with CS polymer for developing electrochemical immune-sensors and electrochemical enzyme biosensors [133]. In the case of optical sensors, Bhatnagar et al. have constructed a nanobiosensor for diagnosis of invasive *Aspergillosis* using CS-stabilized AuNPs. The sensor probe was fabricated using 1,6-Hexanedithiol and CS stabilized AuNPs. The sensor was characterized by SPR absorbance spectra [134]. An excellent work by Wang et al. resulted in fabrication of biosensors based on a combination of CS and Prussian blue dye. The biosensors included a glucose sensor, glutamate sensor, and a galactose sensor. The sensors’ interference with ascorbic acid and uric acid were also selectively analyzed. The biosensors based on a CS platform were able to detect glucose, galactose, and glutamine in human blood serum and in fermented solutions [135]. In 2010, Xia and coworkers developed a novel CdSe-CS hybrid fluorescent film and demonstrated the utilization of a film for selectively sensing the presence of polyamines. The CS polymer was shown to prohibit aggregation of the CsSe micro-nano-particles [136]. In this work, the authors used CS to make hybrid films based on its film-forming and binding ability with heavy metal ions. From PL emission data, the films were shown to selectively sense polyamines. Also, a heavy metal ion sensor, based on the fluorescence of FITC, was successfully prepared and tested by the Lee group [136]. The sensor was constructed from CS oligomer and showed effective detection of copper ions by fluorescence quenching. The CS oligomer–FITC complex was synthesized by simple mixing of FITC with CS oligomer [137]. The hybrid films exhibited blue fluorescence emission, and, with increasing concentration of CS, the PL intensity was enhanced. There have been numerous works on sensing using CS-based plasmonic nanoparticles. A label-free colorimetric sensor for sensing melamine was constructed from CS-stabilized AuNPs by Guan et al. As noticed in Figure 6, the melamine detection was simply based on the aggregation of CS-stabilized AuNPs. The AuNPs were synthesized within CS in the presence of TPP. The CS was used as both the reducing and stabilizing agent. The presence of melamine resulted in a blue shift of the SPR band [138]. Nitrites (NO_2_^−^) are an essential, inorganic, nitrogen-containing nutrient for plant growth. They are also widely used for food preservation and meat curing. In humans, the high concentration of nitrite is known to be associated with medical issues such as gastric cancer and hypertension. In 2017, Amanulla and coworkers reported AuNPs–graphene oxide composites stabilized in CS medium for the first time and applied them for colorimetric detection of NO_2_^−^. Here, CS was used as a stabilizing agent, and succinic acid was used as a reducing agent. The SPR exhibited a red shift upon addition of NO_2_^−^ ions to the solution of AuNPs–graphene oxide composite [139]. Another target for colorimetric detection is the biological macromolecule heparin, because of its important role in the regulation of various physiological and pathological processes. Realizing the significance of heparin, many sensors have been fabricated based on different platforms. CS-stabilized AuNPs were prepared and based on the intensity of scattered light, and the presence vs. absence of heparin was investigated. Addition of small amounts of heparin to CS stabilized AuNPs resulted in an increase in the intensity of a weak resonance light scattering (RLS) [140]. Recently, our group has demonstrated the sensing of amine-based polymers using phosphorescent CS nanoparticles. In this work, we have noticed that the self-assembled phosphorescent CS nanoparticle initially exhibited weak PL intensity, but upon addition of just a few microliters of some selective amines, the PL intensity was dramatically enhanced by an order of magnitude. In this work, we assumed the electrostatic interactions between the amine groups of the polymers and the sulfonate groups of the Au(I) complex were causing the PL enhancement [46]. In a different work published by our group, we have used CS polymer as a platform for stabilizing an optically rich Au(I)-based trinuclear trimer system. The CS stabilized trinuclear trimer system exhibited parts-per-billion (ppb) sensitivity to Ag^+^ ions (Figure 7). In the absence of Ag^+^, the luminescent CS system exhibits red emission at 675 nm, and upon addition of ppb levels of Ag^+^, the emission is blue-shifted to 475 nm and exhibits bright green emission. We hypothesized the formation of luminescent CS due to the interactions between amine groups of CS and carboxylic acid groups of the pyrazole ligand in the complex [70]. Layer by layer (LBL) biosensors films are known for their versatility in immobilizing the sensory material while maintaining their sensory activity. The need for the determination of organophosphates residues is increasing, due to its significance in agriculture, water treatment, environment, and in biology. Recently, organophosphate sensors based on LBL technique were designed using CS and thioglycolic acid stabilized CdSe quantum dots [61]. By comparison, the film-forming ability of CS has been employed for sensing Hg^2+^ using ZnS QD-doped CS films. The luminescent films showed a 15-min response time and 5 ppm detection limit [61]. In another work, Cu^2+^ detection was performed using CS-stabilized gold nanoclusters and a similar chemistry as stated above. These gold nanoclusters were employed and achieved a 1 ppm detection limit with a 10-min reaction time [61].

#### 2.3.3. Gene Therapy

Cancer cells differ from normal cells by the mutation of genes [26]. Cancer gene therapy is a method with which to recognize cancer and treat it at the gene level [26]. Gene therapy can be broken into two main approaches. In the first approach, there is gene augmentation to upregulate tumor suppressor genes [26]. The second approach involves gene knockdown using short, interfering RNA (siRNA) [26]. For successful nanoparticle gene therapy, the therapeutic genes have to be protected from gene cleavage enzymes and transported into the targeted intracellular compartments [26]. Oligonucleotide-coated NPs conjugates are useful for drug delivery, gene therapy, and diagnostics [141]. Kenneth et al. reported that the CS/siRNA nanoparticles enhance the green fluorescent protein gene knockdown in both human lung carcinoma cells and murine peritoneal macrophages [142]. These NP-oligonucleotide structures showed high levels of cellular uptake and transfection efficiency [141]. Moreover, the structure showed resistance against degradation by a nuclease, minimal immune response; low toxicity; and highly effective gene regulating capabilities [141]. Many existing NP-oligonucleotide structures are composed of AuNPs cores and oligonucleotide shells (AuNPs-oligonucleotides) [141]. However, the application of AuNPs is hindered due to the lack of characterization of their long-term toxicity, and the observation that they characteristically show usually high scattering background from the cells by dark field imaging, which limits their applications for cellular imaging [141]. Materials are needed to broaden the application of NP-oligonucleotide beyond gold-based conjugates. Carbon-based materials are an attractive alternative in biomedicine because of tunable fluorescence, high loading efficiency, and convenient preparation [141]. Recently, CDs have been utilized as imaging-guided nanocarriers for the delivery of chemotherapeutics and genes [141]. Unfortunately, these CDs need post-modifications through tedious work [141]. On the positive side, researchers have prepared highly amino functionalized CDs through a one-step, inexpensive, and easy process by hydrothermal reaction with CS [141]. These amino-functionalized CDs have not been previously utilized to load and transport nucleic acids (siRNA) [141]. In response, Zhang et al. synthesized NP-oligonucleotide conjugates with a CS-derived CD core and siRNA shell to explore their function in gene delivery and regulation for tumor therapy [141]. Zhang’s research showed that the dosage of CDs needed to deliver the same amount of siRNA was much lower than that of the AuNPs [141]. In vitro and in vivo experiments demonstrated the excellent performance of the CS-CD with a shell of siRNA in the following areas: entering cancer cells, efficient down-regulation of Plk1 gene (regulates mitosis and is often overexpressed in tumor cells), and potent tumor inhibition effects [141].

## 3. Conclusions and Future Perspectives

In light of the above discussion, it is very clear that CS polymer usage has received tremendous attention in the fields of optically active molecular and nanomaterial systems. Owing to the biologically benign nature of CS, its usage for stabilization of optically active materials (e.g., plasmonic nanoparticles, QDs, luminescent lanthanide nanoparticles, and luminescent transitional organometallic complexes) has resulted in hybrid luminescent systems that are shown to possess better biocompatibility and stability compared to their non-CS counterparts. These hybrid systems were shown to be useful in sensing, imaging, and PTT applications. In the case of plasmonic nanoparticles, the presence of CS has improved the biocompatibility and helped to overcome the environmental and cellular toxicity concerns allowing for in vitro and in vivo applications of gold and silver nanoparticles. This reduced toxicity is due to CS’ ability to be a solvent, a reducing agent, and a stabilizing agent, which has helped to satisfy the requirements of “green” chemistry methods. The use of CS for stabilizing visible absorbing gold and silver nanospheres is extensively exploited compared to the synthesis of NIR absorbing anisotropic nanoparticles. Anisotropic gold and silver nanoparticles are more intriguing for their optical properties and more challenging to synthesize compared to nanospheres. Applying CS as a stabilizing agent for such NPs is highly effective, because it avoids the use of a toxic surfactant like CTAB. With the very limited number of research papers published on the usage of CS for making NIR absorbing plasmonic NPs, there are significant opportunities still available for researchers to explore in this particular area. In case of luminescent systems, the use of CS as stabilizing or capping agent is extensively demonstrated in QD systems compared to luminescent complexes. In QDs, the presence of CS has helped with water solubility, chemical stability, and compatibility with physiological media. Some of these CS capped QDs have been shown to overcome challenges of biological stability for deep tissue imaging, while the film-forming ability of CS has been employed for making luminescent films that are shown to be active for sensing heavy and toxic metals. In the case of luminescent organometallic molecular systems, there are some advanced works, including the development of hypoxia-sensitive phosphorescent CS nanoparticles for tumor imaging and for developing reversible temperature sensors. However, the extent of usage of CS is limited compared to the enormous field of luminescent organometallic complexes. Recently, our group demonstrated the use of CS for stabilizing an Au(I)-based phosphorescent trinuclear trimer complex in aqueous-polymer media. Though our system is similar to other Au(I)-based trimer systems that have been investigated for decades for their rich optoelectronic applications; the utilization of polymers like CS for stabilizing them in polymer-aqueous media has never been attempted. Making such complexes in polymer-aqueous media will extend their applicability to biologically related fields. The area of inorganic-organic systems comprised of luminescent materials and plasmonic nanoparticles is very extensive and widespread. Summarizing all works involving CS polymer under one single review is difficult. Our motive with this review was to “shine” light on CS by highlighting some of the optically active photonic systems made including: QDs, selective organometallic complexes, and silver and gold plasmonic nanoparticles. However, other fascinating plasmonic systems like copper nanoparticles, platinum, palladium, Au-Ag bimetallic nanoparticles, core-shell nanoparticles of gold, silver, and gold-silver, to name a few, are not covered in this review. These systems are very captivating for their interesting chemistries and extensive, non-optical applications. However, solubility or dispersion in aqueous media, leakage of heavy metal ions, aggregation, surface sensitivity, involvement of organic solvents, high temperature treatments, surface ligands for biological work, poor photostability, and compatibility to physiological media are still regarded as crucial issues to overcome in order to realize the full potential of very promising, strongly emissive luminescent dyes and plasmonic nanoparticles for delivery, diagnosis, imaging, and sensing applications. Based on our literature search, we believe that optical chemists dealing with luminescent compounds and plasmonic nanoparticles have not utilized the extraordinary potential of CS to its fullest.

## Figures and Tables

**Table 1:** Some selective CS based fluorescent systems and their applications.

Fluorescent CS Systems	Applications	References
Carbon dots	Bioimaging Photothermal therapy Gene therapy	[75,76]
Quantum dots	Bioimaging in vitro drug delivery Deep tissue imaging Bioconjugation to biomolecules	[44,50,55]
AIE nanoparticles	Sensing Bioimaging	[67]
Fluorescent CS nanoparticles	Specific targeting Cellular imaging Probes Cell uptake and imaging Bioconjugation Chemotherapy Gene therapy	[40,41,44,52,53]
Au/Ag fluorescent nanoclusters	Bioimaging Chemo and biosensing Luminescent films for sensing	[51,61,68,69]
Luminescent organometallic complexes	Temperature sensing Hypoxia sensing Silver metal sensing Sensing small molecules Eradication of carcinoma cells Humidity sensors Determination of heavy metals (Hg^2+^, Ag^+^, Cu^2+^, etc.)	[46,56,70,71,72,77]

**Table 2:** Selective applications of gold (AuNPs) and silver (AgNPs) nanoparticles.

Selective Applications of AuNPs & AgNPs	Comments	References
Calorimetric detection by AuNPs	Nanoplasmonic molecular ruler by AuNPs decorated with DNA	[71,72]
Sensing biomolecules by AuNPs	Polyaniline stabilized AuNPs	[72,96]
Detection by SERS (surface-enhanced Raman scattering) of AuNPs	Probing different biomolecules of DNA or nucleic acids or differentiating tumor cells	[97]
Detection by FRET (fluorescence resonance energy transfer) of AuNPs	Detect analyte concentration by quenching	[72]
Sensing by SPR of AuNPs	Presence of analyte by a change in color	[79]
Hyperthermia or plasmonic photothermal therapy using AuNPs	Kill cancer cells The opening of polymeric capsules Melting of DNA or protein bonds Photoacoustic tomography Photothermal imaging	[97,98]
AuNPs as contrast agents	Using antibody labeled AuNPs (binding to anti-epidermal growth factor (EFGR)) Detecting cancer cells using a different optical imaging technique	[80,89]
Immunostaining by AuNPs	Visualization of cellular organelles using simple optical microscopy	[98]
Delivery by AuNPs	Genes DNA Nucleotides or biomolecules either by specific or nonspecific uptake (site specific receptors)	[72,96]
Catalysis by AuNPs	Prepared by physical deposition techniques	[99]
Differentiating tumor cells vs. normal cells by molecular imaging using AuNPs	Darkfield optical imaging of cancer cells Detection of bioconjugated cancer cells using enhanced Raman signals Detection of tumors in deep tissue using two-Photon enhanced luminescence	[100]
Delivery agents (AuNPs)	Multifunctional nanorods for gene delivery Release of plasmids DNA (by laser irradiation)	[101]
Diagnostic applications of silver nanoparticles	Biosensors for tagging and quantitative detection	[102]
Antibacterial applications of AgNPs	Garments Apparels Paints Wound-dressing gels Skin creams Industrial appliances for antibacterial properties	[102]
Conductive applications of AgNPs	Formation of conductive inks Enhances the thermal and electrical conductivity of electrical and optical materials	[103,104]
Optical applications of AgNPs	Detection and sensing using metal-enhanced fluorescence Detection and sensing using surface-enhanced Raman scattering	[103,104]

**Table 3:** Selective methods for synthesis of CS-stabilized gold (AuNPs) and silver nanoparticles (AgNPs) and their corresponding applications.

Chitosan Stabilized AuNPs and AgNPs	Property and/or Applications	References
CS-stabilized AuNPs	Demonstrating formation of AuNPs in absence of a reducing agent	[117]
Stabilization of Au and AgNPs within CS	Adopting/demonstrating “green” synthesis method for making AuNPs and AgNPs	[14]
Synthesis of positively charged AuNPs and negatively charged AgNPs	Adopting/demonstrating “green” synthesis method for making AuNPs and AgNPs	[14]
		
CS-coated AuNPs	Evaluating stability of different size AuNPs with respect to CS molecular weight and concentration	[119]
CS-stabilized AuNPs in presence of TPP	Effect of TPP and CS concentration on size and shape of AuNPs demonstrates AuNPs formation without any additional reducing agents	[117]
CS-capped AuNPs	Sensing heavy metal ions based on SPR changes	[121]
Gold–CS nanocomposites	Selective electrochemical sensors for the determination of antioxidants. Determination of polyphenol index in wines	[122]
CS-embedded AuNPs	As a substrate for SERS	[118]
CS–PAA–Au hybrid nanospheres via the one-pot route in aqueous media	Contrast agents and delivery agents	[123]
Surface functionalization of AuNRs with CS oligosaccharides	Provides multiple binding sites for robust coating and protection against aggregation and as delivery agents	[124]
CS-stabilized plasmonic nanoparticle	Penetration and uptake of therapeutic agents such as insulin across the mucosal membrane	[109]
CS-stabilized AgNPs	Enhance antibacterial activity and overcome concerns about human and environmental safety related to usage of these metal nanoparticles	[109]
CS-stabilized silver nanoparticles in presence of cotton fabric	Antibacterial activity of cotton fabrics	[113]
Silk fibroin/carboxymethyl, CS-stabilized AgNPs	Wound healing/wound dressing application	[111,112]
CS-stabilized NIR absorbing, anisotropic AgNPs	Demonstration of a photochemical method for stabilizing NIR AgNPs and their antipathogenic properties	[114]
CS-stabilized, anisotropic AgNPs	Substrate for SERS and single molecule detection	[115]
CS-stabilized nanotriangles	Novel biocompatible and highly effective photothermal transducers for in vitro cancer cell therapy	[116]
CS–Siloxane cross-linked silver nanocomposites	Enhanced antibacterial properties	[110]
CS-stabilized AgNPs—understanding effect of molecular weight of CS on the size of AgNPs	Antibacterial activity against *Staphylococcus aureus*	[13]
Synthesis of silver/CS/polyethylene glycol nanocomposites	“Green” synthesis methodology, understanding the effect of temperature on the size of AgNPs	[15]

## Abbreviations

CS	Chitosan
TPP	Tripolyphosphate
LBL	Layer by layer
FITC	Fluorescein isothiocyanate
ZnONPs	Zinc oxide nanoparticles
ROS	Reactive oxygen species
MW	Molecular weight
FCSNPs	Fluorescent chitosan nanoparticles
CPT	Camptothecin
PEC	Polyelectrolyte complexation
CCHNs	Chitosan–carbon dot hybrid nanogels
SERS	Surface-enhanced Raman spectroscopy
MEF	Metal-enhanced fluorescence
SPA	Surface plasmon resonance
PL	Photoluminescence
LPNs	Luminescent polymeric nanoparticles
NIRF	Near-infrared fluorescent
ACQ	Aggregation-caused quenching
AIEgens	Aggregated-induced emission luminogens
NPs	Nanoparticles
AuNPs	Gold nanoparticles
AgNPs	Silver nanoparticles
QDs	Quantum dots
CDs	Carbon dots
AIE	Aggregated-induced emission

## References

[1] Hudson S.M., Jenkins D.W. (2001). Encyclopedia of Polymer Science and Technology.

[2] Ruel-Gariѐpy E., Leroux J. (2006). Chitosan: A Natural Polycation with Multiple Applications.

[3] Jain A., Gulbake A., Shilpi S., Jain A., Hurkat P., Jain S.K. (2013). A New Horizon in Modifications of Chitosan: Syntheses and Applications. Crit. Rev. Ther. Drug Carr. Syst..

[4] Ravi Kumar M.N.V. (2000). A Review of Chitin and Chitosan Applications. React. Funct. Polym..

[5] Wang X., Luo Y., Li X., Ling Y., Shen Z., Han G., Sun R. (2013). Nanocomposites: Synthesis, Characterization and Applications.

[6] Sanford P.A., Hutchings G.P. (1987). Industrial Polysaccharides: Genetic Engineering, Structure/Property Relations, and Applications.

[7] Sivakumar S.M., Kannadasan M., Roy R.K. (2014). Review of Chitosan and its Relevance in Pharmaceutical Sciences. Res. J. Pharm. Biol. Chem. Sci..

[8] Agrawal P., Strijkers G.J., Nicolay K. (2010). Chitosan-Based Systems for Molecular Imaging. Adv. Drug Deliv. Rev..

[9] Wang Y., Zhang Q., Zhang C., Li P. (2012). Characterisation and Cooperative Antimicrobial Properties of Chitosan/Nano-ZnO Composite Nanofibrous Membranes. Food Chem..

[10] Fei L.X., Lin G.Y., Zhi Y.D., Zhi L., De Y.K. (2000). Antibacterial Action of Chitosan and Carboxymethylated Chitosan. J. Appl. Polym. Sci..

[11] Sudarshan N.R., Hoover D.G., Knorr D. (1992). Antibacterial Action of Chitosan. Food Biotechnol..

[12] Kong M., Chen X.G., Xing K., Park H.J. (2010). Antimicrobial Properties of Chitosan and Mode of Action: A State of the Art Review. Int. J. Food. Microbiol..

[13] Honary S., Ghajar K.A., Khazaeli P., Shalchian P. (2011). Preparation, Characterization and Antibacterial Properties of Silver-Chitosan Nanocomposites using Different Molecular Weight Grades of Chitosan. Trop. J. Pharm. Res..

[14] Huang H., Yang X. (2004). Synthesis of Polysaccharide-Stabilized Gold and Silver Nanoparticles: A Green Method. Carbohydr. Res..

[15] Ahmad M., Tay M., Shameli K., Hussein M., Lim J. (2011). Green Synthesis and Characterization of Silver/Chitosan/Polyethylene Glycol Nanocomposites without any Reducing Agent. Int. J. Mol. Sci..

[16] Xing R., Yu H., Liu S., Zhang W., Zhang Q., Li Z., Li P. (2005). Antioxidant Activity of Differently Regioselective Chitosan Sulfates in Vitro. Bioorg. Med. Chem..

[17] Sousa F., Guebitz G.M., Kokol V. (2009). Antimicrobial and Antioxidant Properties of Chitosan Enzymatically Functionalized with Flavonoids. Process Biochem..

[18] Vinsova J., Vavrikova E. (2011). Chitosan Derivatives with Antimicrobial, Antitumour and Antioxidant Activities—A Review. Curr. Pharm. Des..

[19] Guo Z., Xing R., Liu S., Zhong Z., Li P. (2008). Synthesis and Hydroxyl Radicals Scavenging Activity of Quaternized Carboxymethyl Chitosan. Carbohydr. Polym..

[20] Montemor M.F. (2014). Functional and Smart Coatings for Corrosion Protection: A Review of Recent Advances. Surf. Coat. Technol..

[21] Carneiro J., Tedim J., Ferreira M.G.S. (2015). Chitosan as a Smart Coating for Corrosion Protection of Aluminum Alloy 2024: A Review. Prog. Org. Coat..

[22] Carneiro J., Tedim J., Fernandes S.C.M., Freire C.S.R., Silvestre A.J.D., Gandini A., Ferreira M.G.S., Zheludkevich M.L. (2012). Chitosan-Based Self-Healing Protective Coatings Doped with Cerium Nitrate for Corrosion Protection of Aluminum Alloy 2024. Prog. Org. Coat..

[23] Ghosh B., Urban M.W. (2009). Self-Repairing Oxetane-Substituted Chitosan Polyurethane Networks. Science.

[24] Elgadir M.A., Uddin M.S., Ferdosh S., Adam A., Chowdhury A.J.K., Sarker M.Z.I. (2015). Impact of Chitosan Composites and Chitosan Nanoparticle Composites on various Drug Delivery Systems: A Review. J. Food Drug Anal..

[25] Kumar N., Patel A.K., Kumari N., Kumar A. (2014). A Review on Chitosan Nanoparticles for Cancer Treatment. Int. J. Nanomater. Biostruct..

[26] Key J., Park K. (2017). Multicomponent, Tumor-Homing Chitosan Nanoparticles for Cancer Imaging and Therapy. Int. J. Mol. Sci..

[27] Min K.H., Park K., Kim Y., Bae S.M., Lee S., Jo H.G., Park R., Kim I., Jeong S.Y., Kim K. (2008). Hydrophobically Modified Glycol Chitosan Nanoparticles-Encapsulated Camptothecin Enhance the Drug Stability and Tumor Targeting in Cancer Therapy. J. Controll. Release.

[28] Kim K., Kim J.H., Park H., Kim Y., Park K., Nam H., Lee S., Park J.H., Park R., Kim I. (2010). Tumor-Homing Multifunctional Nanoparticles for Cancer Theragnosis: Simultaneous Diagnosis, Drug Delivery, and Therapeutic Monitoring. J. Controll. Release.

[29] Ravi Kumar M.N.V., Muzzarelli R.A.A., Muzzarelli C., Sashiwa H., Domb A.J. (2004). Chitosan Chemistry and Pharmaceutical Perspectives. Chem. Rev..

[30] Souza V.G.L., Fernando A.L. (2016). Nanoparticles in Food Packaging: Biodegradability and Potential Migration to food—A Review. Food Packag. Shelf Life.

[31] Ferreira A.R.V., Alves V.D., Coelhoso I.M. (2016). Polysaccharide-Based Membranes in Food Packaging Applications. Membranes.

[32] No H.K., Meyers S.P., Prinyawiwatkul W., Xu Z. (2007). Applications of Chitosan for Improvement of Quality and Shelf Life of Foods: A Review. J. Food Sci..

[33] Ahn D.H., Choi J.S., Lee H.Y., Kim J.Y., Youn S.K., Park S.M. (2003). Effects on Preservation and Quality of Bread with Coating High Molecular Weight Chitosan. Korean J. Food Nutr..

[34] Ghaouth A., Arul J., Ponnampalam R., Boulet M. (2006). Chitosan Coating Effect on Storability and Quality of Fresh Strawberries. J. Food Sci..

[35] Zhang L., Zeng Y., Cheng Z. (2016). Removal of Heavy Metal Ions using Chitosan and Modified Chitosan: A Review. J. Mol. Liq..

[36] Bhatnagar A., Sillanpää M. (2009). Applications of Chitin- and Chitosan-Derivatives for the Detoxification of Water and Wastewater—A Short Review. Adv. Colloid Interface Sci..

[37] Pontoni L., Fabbricino M. (2012). Use of Chitosan and Chitosan-Derivatives to Remove Arsenic from Aqueous Solutions—A Mini Review. Carbohydr. Res..

[38] Asghari F., Samiei M., Adibkia K., Akbarzadeh A., Davaran S. (2017). Biodegradable and Biocompatible Polymers for Tissue Engineering Application: A Review. Artif. Cells Blood Substit. Biotechnol..

[39] Rodríguez-Vázquez M., Vega-Ruiz B., Ramos-Zúñiga R., Saldaña-Koppel D.A., Quiñones-Olvera L.F. (2015). Chitosan and its Potential use as a Scaffold for Tissue Engineering in Regenerative Medicine. BioMed Res. Int..

[40] Ge Y., Zhang Y., He S., Nie F., Teng G., Gu N. (2009). Fluorescence Modified Chitosan-Coated Magnetic Nanoparticles for High-Efficient Cellular Imaging. Nanoscale Res. Lett..

[41] Huang M., Ma Z., Khor E., Lim L. (2002). Uptake of FITC-Chitosan Nanoparticles by A549 Cells. Pharm. Res..

[42] Bui V., Park D., Lee Y. (2017). Chitosan Combined with ZnO, TiO_2_ and Ag Nanoparticles for Antimicrobial Wound Healing Applications: A Mini Review of the Research Trends. Polymers.

[43] Nie Q., Tan W.B., Zhang Y. (2006). Synthesis and Characterization of Monodisperse Chitosan Nanoparticles with Embedded Quantum Dots. Nanotechnology.

[44] Tian Y., Yu J., Qi X., Wu X., Hua R., Fan S. (2009). Bio-Conjugation of CaF_2_:Eu/Chitosan Nanoparticles with BSA and Photoluminescent Properties. J. Mater. Sci. Mater. Electron..

[45] Wu H., Zhang J. (2018). Chitosan-Based Zinc Oxide Nanoparticle for Enhanced Anticancer Effect in Cervical Cancer: A Physicochemical and Biological Perspective. Saudi Pharm. J..

[46] Marpu S., Upadhyay P.K., Nguyen D.T., Oswald I.W.H., Arvapally R.K., Petros R.A., Hu Z., Omary M.A. (2015). Self-Assembly of Linear Polymers into Phosphorescent Nanoparticles: Optimization Toward Non-Cytotoxic Bioimaging and Photonic Devices. J. Phys. Chem. C.

[47] Santra S., Dutta D., Walter G.A., Moudgil B.M. (2005). Fluorescent Nanoparticle Probes for Cancer Imaging. Technol. Cancer Res. Treat..

[48] Lucky S.S., Soo K.C., Zhang Y. (2015). Nanoparticles in Photodynamic Therapy. Chem. Rev..

[49] Berger J., Reist M., Mayer J.M., Felt O., Gurny R. (2004). Structure and Interactions in Chitosan Hydrogels Formed by Complexation or Aggregation for Biomedical Applications. Eur. J. Pharm. Biopharm..

[50] Yuan Q., Hein S., Misra R.D.K. (2010). New Generation of Chitosan-Encapsulated ZnO Quantum Dots Loaded with Drug: Synthesis, Characterization and in Vitro Drug Delivery Response. Acta Biomater..

[51] Duan Y., Duan R., Liu R., Guan M., Chen W., Ma M., Du B., Zhang Q. (2018). Chitosan-Stabilized Self-Assembled Fluorescent Gold Nanoclusters for Cell Imaging and Biodistribution in Vivo. ACS Biomater. Sci. Eng..

[52] Tallury P., Kar S., Bamrungsap S., Huang Y., Tan W., Santra S. (2009). Ultra-Small Water-Dispersible Fluorescent Chitosan Nanoparticles: Synthesis, Characterization and Specific Targeting. Chem. Commun..

[53] Zhao J., Wu J. (2006). Preparation and Characterization of the Fluorescent Chitosan Nanoparticle Probe. Chin. J. Anal. Chem..

[54] Wang F., Zhang Y., Fan X., Wang M. (2006). One-Pot Synthesis of Chitosan/LaF_3_ :Eu^3+^ Nanocrystals for Bio-Applications. Nanotechnology.

[55] Sandros M.G., Behrendt M., Maysinger D., Tabrizian M. (2007). InGaP@ZnS-Enriched Chitosan Nanoparticles: A Versatile Fluorescent Probe for Deep-Tissue Imaging. Adv. Funct. Mater..

[56] Zeng Y., Zhang S., Jia M., Liu Y., Shang J., Guo Y., Xu J., Wu D. (2013). Hypoxia-Sensitive Bis(2-(2′-Benzothienyl)Pyridinato-*N*,*C*^3′^)Iridium[Poly(N-Butyl Cyanoacrylate]/Chitosan Nanoparticles and their Phosphorescence Tumor Imaging in Vitro and in Vivo. Nanoscale.

[57] Chen M., Yin M. (2014). Design and Development of Fluorescent Nanostructures for Bioimaging. Prog. Polym. Sci..

[58] Ghormade V., Gholap H., Kale S., Kulkarni V.M., Bhat S., Paknikar K.M. (2015). Fluorescent Cadmium Telluride Quantum Dots Embedded Chitosan Nanoparticles: A Stable, Biocompatible Preparation for Bio-Imaging. J. Biomater. Sci. Polym. Ed..

[59] Salgado C.L., Mansur A.A.P., Mansur H.S., Monteiro F.J.M. (2014). Fluorescent Bionanoprobes Based on Quantum Dot-Chitosan-O-Phospho-L-Serine Conjugates for Labeling Human Bone Marrow Stromal Cells. RSC Adv..

[60] Wolfbeis O.S. (2015). An Overview of Nanoparticles Commonly used in Fluorescent Bioimaging. Chem. Soc. Rev..

[61] Guan W., Zhou W., Lu J., Lu C. (2015). Luminescent Films for Chemo- and Biosensing. Chem. Soc. Rev..

[62] Song Y., Zhu S., Yang B. (2014). Bioimaging Based on Fluorescent Carbon Dots. RSC Adv..

[63] Yang Y., Cui J., Zheng M., Hu C., Tan S., Xiao Y., Yang Q., Liu Y. (2012). One-Step Synthesis of Amino-Functionalized Fluorescent Carbon Nanoparticles by Hydrothermal Carbonization of Chitosan. Chem. Commun..

[64] Wan Q., Liu M., Xu D., Mao L., Tian J., Huang H., Gao P., Deng F., Zhang X., Wei Y. (2016). Fabrication of Aggregation Induced Emission Active Luminescent Chitosan Nanoparticles Via a “one-Pot” Multicomponent Reaction. Carbohydr. Polym..

[65] Chen S., Wang H., Hong Y., Tang B.Z. (2016). Fabrication of Fluorescent Nanoparticles Based on AIE Luminogens (AIE Dots) and their Applications in Bioimaging. Mater. Horiz..

[66] Xie G., Ma C., Zhang X., Liu H., Yang L., Li Y., Wang K., Wei Y. (2017). Chitosan-Based Cross-Linked Fluorescent Polymer Containing Aggregation-Induced Emission Fluorogen for Cell Imaging. Dyes Pigm..

[67] He G., Peng H., Liu T., Yang M., Zhang Y., Fang Y. (2009). A Novel Picric Acid Film Sensor Via Combination of the Surface Enrichment Effect of Chitosan Films and the Aggregation-Induced Emission Effect of Siloles. J. Mater. Chem..

[68] Binnemans K. (2009). Lanthanide-Based Luminescent Hybrid Materials. Chem. Rev..

[69] Wang C., Huang Y. (2014). Facile Preparation of Fluorescent Ag-Clusters-Chitosan-Hybrid Nanocomposites for Bio-Applications. New J. Chem..

[70] Upadhyay P.K., Marpu S.B., Benton E.N., Williams C.L., Telang A., Omary M.A. (2018). A Phosphorescent Trinuclear Gold(I) Pyrazolate Chemosensor for Silver Ion Detection and Remediation in Aqueous Media. Anal. Chem..

[71] Tsvirko M., Tkaczyk S., Kozak M., Kalota B. (2013). Luminescent Temperature Sensor Based on Ru(Bpy)_3_]^2+^ Incorporated into Chitosan. Funct. Mater..

[72] Bustamante N., Lelasi G., Bedoya M., Orellana G. (2018). Optimization of Temperature Sensing with Polymer-Embedded Luminescent Ru(II) Complexes. Polymers.

[73] Takato K., Gokan N., Kaneko M. (2005). Effect of Humidity on Photoluminescence from Ru(Bpy)_3_^2+^ Incorporated into a Polysaccharide Solid Film and its Application to Optical Humidity Sensor. J. Photochem. Photobiol. A Chem..

[74] Chakraborty I., Jimenez J., Mascharak P.K. (2017). CO-Induced Apoptotic Death of Colorectal Cancer Cells by a Luminescent photoCORM Grafted on Biocompatible Carboxymethyl Chitosan. Chem. Commun..

[75] Wang H., Mukherjee S., Yi J., Banerjee P., Chen Q., Zhou S. (2017). Biocompatible Chitosan–Carbon Dot Hybrid Nanogels for NIR-Imaging-Guided Synergistic Photothermal–Chemo Therapy. ACS Appl. Mater. Interfaces.

[76] Song X., Wu H., Li S., Wang Y., Ma X., Tan M. (2015). Ultrasmall Chitosan–Genipin Nanocarriers Fabricated from Reverse Microemulsion Process for Tumor Photothermal Therapy in Mice. Biomacromolecules.

[77] Lo K. (2006). Photofunctional Transition Metal Complexes.

[78] Daniel M., Astruc D. (2004). Gold Nanoparticles: Assembly, Supramolecular Chemistry, Quantum-Size-Related Properties, and Applications toward Biology, Catalysis, and Nanotechnology. Chem. Rev..

[79] Eustis S., El-Sayed M.A. (2006). Why Gold Nanoparticles are More Precious than Pretty Gold: Noble Metal Surface Plasmon Resonance and its Enhancement of the Radiative and Nonradiative Properties of Nanocrystals of Different Shapes. Chem. Soc. Rev..

[80] Kim D., Park S., Lee J.H., Jeong Y.Y., Jon S. (2007). Antibiofouling Polymer-Coated Gold Nanoparticles as a Contrast Agent for in Vivo X-Ray Computed Tomography Imaging. J. Am. Chem. Soc..

[81] Mandal R., Mandal D., Mishra N., Bahadur A. (2010). Effect of Surfactants on Phosphatase Level of Fresh Water Fish Labeo Rohita. J. Environ. Biol..

[82] Bindu P.C., Babu P. (2001). Surfactant-Induced Lipid Peroxidation in a Tropical Euryhaline Teleost Oreochromis Mossambicus (Tilapia) Adapted to Fresh Water. Indian. J. Exp. Biol..

[83] Alkilany A., Murphy C. (2010). Toxicity and Cellular Uptake of Gold Nanoparticles: What we have Learned so Far?. J. Nanopart. Res..

[84] Schmid G., Corain B. (2003). Nanoparticulated Gold: Syntheses, Structures, Electronics, and Reactivities. Eur. J. Inorg. Chem..

[85] Tolaymat T.M., El Badawy A.M., Genaidy A., Scheckel K.G., Luxton T.P., Suidan M. (2010). An Evidence-Based Environmental Perspective of Manufactured Silver Nanoparticle in Syntheses and Applications: A Systematic Review and Critical Appraisal of Peer-Reviewed Scientific Papers. Sci. Total Environ..

[86] Raveendran P., Fu J., Wallen S.L. (2003). Completely “Green” Synthesis and Stabilization of Metal Nanoparticles. J. Am. Chem. Soc..

[87] Lu L., Ai K., Ozaki Y. (2008). Environmentally Friendly Synthesis of Highly Monodisperse Biocompatible Gold Nanoparticles with Urchin-Like Shape. Langmuir.

[88] Sur I., Cam D., Kahraman M., Baysal A., Culha M. (2010). Interaction of Multi-Functional Silver Nanoparticles with Living Cells. Nanotechnology.

[89] Shan J., Tenhu H. (2007). Recent Advances in Polymer Protected Gold Nanoparticles: Synthesis, Properties and Applications. Chem. Commun..

[90] Ahmed S., Ahmad M., Swami B.L., Ikram S. (2016). A Review on Plants Extract Mediated Synthesis of Silver Nanoparticles for Antimicrobial Applications: A Green Expertise. J. Adv. Res..

[91] Ahmed S., Ikram S. (2015). Synthesis of Gold Nanoparticles using Plant Extract: An Overview. Nano Res. Appl..

[92] Pasca R., Mocanu A., Cobzac S., Petean I., Horovitz O., Tomoaia-Cotisel M. (2014). Biogenic Syntheses of Gold Nanoparticles using Plant Extracts. Part. Sci. Technol..

[93] Sharma G., Jasuja N.D., Kumar M., Ali M.I. (2015). Biological Synthesis of Silver Nanoparticles by Cell-Free Extract of Spirulina Platensis. J. Nanotechnol..

[94] Rashid Z., Moadi T., Ghahremanzadeh R. (2016). Green Synthesis and Characterization of Silver Nanoparticles using *Ferula Latisecta* Leaf Extract and their Application as a Catalyst for the Safe and Simple One-Pot Preparation of Spirooxindoles in Water. New J. Chem..

[95] Phukan A., Chetia B. (2015). Green Synthesis, Catalytic and Antibacterial Activity of Silver Nanoparticles Synthesized from Olax Acuminata. Asian J. Chem..

[96] Xian Y., Hu Y., Liu F., Xian Y., Wang H., Jin L. (2006). Glucose Biosensor Based on Au Nanoparticles–conductive Polyaniline Nanocomposite. Biosens. Bioelectron..

[97] Fazio B., D’Andrea C., Foti A., Messina E., Irrera A., Donato M.G., Villari V., Micali N., Marago O., Gucciardi P. (2016). SERS Detection of Biomolecules at Physiological pH Via Aggregation of Gold Nanorods Mediated by Optical Forces and Plasmonic Heating. Sci. Rep..

[98] Xiaohua H., Jain P.K., El-Sayed I.H., El-Sayed M.A. (2007). Determination of the Minimum Temperature Required for Selective Photothermal Destruction of Cancer Cells with the use of Immunotargeted Gold Nanoparticles. Photochem. Photobiol..

[99] Fierro-Gonzalez J.C., Guzman J., Gates B.C. (2007). Role of Cationic Gold in Supported CO Oxidation Catalysts. Top. Catal..

[100] Mallidi S., Kim S., Karpiouk A., Joshi P.P., Sokolov K., Emelianov S. (2015). Visualization of Molecular Composition and Functionality of Cancer Cells using Nanoparticle-Augmented Ultrasound-Guided Photoacoustics. Photoacoustics.

[101] Murphy C.J., Sau T.K., Gole A.M., Orendorff C.J., Gao J., Gou L., Hunyadi S.E., Li T. (2005). Anisotropic Metal Nanoparticles: Synthesis, Assembly, and Optical Applications. J. Phys. Chem. B.

[102] Ge L., Li Q., Ouyang J., Li X., Xing M.M. (2014). Nanosilver Particles in Medical Applications: Synthesis, Performance, and Toxicity. Int. J. Nanomed..

[103] Aslan K., Lakowicz J.R., Geddes C.D. (2005). Metal-Enhanced Fluorescence using Anisotropic Silver Nanostructures: Critical Progress to Date. Anal. Bioanal. Chem..

[104] Tien H., Huang Y., Yang S., Wang J., Ma C.M. (2011). The Production of Graphene Nanosheets Decorated with Silver Nanoparticles for use in Transparent, Conductive Films. Carbon.

[105] Chaloupka K., Malam Y., Seifalian A.M. (2010). Nanosilver as a New Generation of Nanoproduct in Biomedical Applications. Trends Biotechnol..

[106] Salomoni R., Léo P., Montemor A.F., Rinaldi B.G., Rodrigues M.F.A. (2017). Antibacterial Effect of Silver Nanoparticles in *Pseudomonas Aeruginosa*. Nanotechnol. Sci. Appl..

[107] Haider S.Z., Kiran U., Ali M.I., Jamal A., Hameed A., Ahmed S., Ali N. (2013). Combined Efficacy of Biologically Synthesized Silver Nanoparticles and Different Antibiotics Against Multidrug-Resistant Bacteria. Int. J. Nanomed..

[108] Sriram M.I., Kanth S.B.M., Kalishwaralal K., Gurunathan S. (2010). Antitumor Activity of Silver Nanoparticles in Dalton’s Lymphoma Ascites Tumor Model. Int. J. Nanomed..

[109] Ahmed T., Aljaeid B. (2016). Preparation, Characterization, and Potential Application of Chitosan, Chitosan Derivatives, and Chitosan Metal Nanoparticles in Pharmaceutical Drug Delivery. Drug Des. Dev. Ther..

[110] Ryan C., Alcock E., Buttimer F., Schmidt M., Clarke D., Pemble M., Bardosova M. (2017). Synthesis and Characterisation of Cross-Linked Chitosan Composites Functionalised with Silver and Gold Nanoparticles for Antimicrobial Applications. Sci. Technol. Adv. Mater..

[111] Rinehart S.J., Campbell T.D., Burke K.J., Garcia B.C., Mlynarski A., Brain S.J., Truffa J.M., Rago J., Chura W.E., Keleher J.J. (2016). Synthesis and Characterization of a Chitosan/PVA Antimicrobial Hydrogel Nanocomposite for Responsive Wound Management Materials. J. Microb. Biochem. Technol..

[112] Pei Z., Sun Q., Sun X., Wang Y., Zhao P. (2015). Preparation and Characterization of Silver Nanoparticles on Silk Fibroin/Carboxymethylchitosan Composite Sponge as Anti-Bacterial Wound Dressing. Biomed. Mater. Eng..

[113] Hien N., Phu D., Duy N., Anh Quoc L., Kim Lan N.T., Hoang D., Hong Van H.T., Diem H.N., Thai Hoa T. (2015). Influence of Chitosan Binder on the Adhesion of Silver Nanoparticles on Cotton Fabric and Evaluation of Antibacterial Activity. Adv. Nanopart..

[114] Marpu S., Kolailat S.S., Korir D., Kamras B.L., Chaturvedi R., Joseph A., Smith C.M., Palma M.C., Shah J., Omary M.A. (2017). Photochemical Formation of Chitosan-Stabilized Near-Infrared-Absorbing Silver Nanoworms: A “Green” Synthetic Strategy and Activity on Gram-Negative Pathogenic Bacteria. J. Colloid Interface Sci..

[115] Potara M., Baia M., Farcau C., Astilean S. (2012). Chitosan-Coated Anisotropic Silver Nanoparticles as a SERS Substrate for Single-Molecule Detection. Nanotechnology.

[116] Boca S.C., Potara M., Gabudean A., Juhem A., Baldeck P.L., Astilean S. (2011). Chitosan-Coated Triangular Silver Nanoparticles as a Novel Class of Biocompatible, Highly Effective Photothermal Transducers for in Vitro Cancer Cell Therapy. Cancer Lett..

[117] Huang H., Yang X. (2004). Synthesis of Chitosan-Stabilized Gold Nanoparticles in the Absence/Presence of Tripolyphosphate. Biomacromolecules.

[118] Potara M., Maniu D., Astilean S. (2009). The Synthesis of Biocompatible and SERS-Active Gold Nanoparticles using Chitosan. Nanotechnology.

[119] Lupusoru R.V., Simion L., Sandu I., Pricop D.A., Chiriac A.P., Poroch V. (2017). Aging Study of Gold Nanoparticles Functionalized with Chitosan in Aqueous Solutions. Rev. Chim..

[120] Le T.L., Dinh Q.K., Tran T.H., Nguyen H.P., Hoang T.L.H., Nguyen Q.H. (2014). Synthesis of Water Soluble Chitosan Stabilized Gold Nanoparticles and Determination of Uric Acid. Adv. Nat. Sci. Nanosci. Nanotechnol..

[121] Sugunan A., Thanachayanont C., Dutta J., Hilborn J.G. (2005). Heavy-Metal Ion Sensors using Chitosan-Capped Gold Nanoparticles. Sci. Technol. Adv. Mater..

[122] Curulli A., Di Carlo G., Ingo G.M., Riccucci C., Zane D., Bianchini C. (2012). Chitosan Stabilized Gold Nanoparticle-Modified Au Electrodes for the Determination of Polyphenol Index in Wines: A Preliminary Study. Electroanalysis.

[123] Hu Y., Chen Q., Ding Y., Li R., Jiang X., Liu B. (2009). Entering and Lighting Up Nuclei using Hollow Chitosan–Gold Hybrid Nanospheres. Adv. Mater..

[124] Erathodiyil N., Jana N.R., Ying J.Y. (2008). Functionalization of Gold Nanospheres and Nanorods by Chitosan Oligosaccharide Derivatives. Adv. Mater..

[125] Ofir Y., Samanta B., Rotello V.M. (2008). Polymer and Biopolymer Mediated Self-Assembly of Gold Nanoparticles. Chem. Soc. Rev..

[126] Uehara N. (2010). Polymer-Functionalized Gold Nanoparticles as Versatile Sensing Materials. Anal. Sci..

[127] Bajpai S., Yallapu M., Bajpai M., Tankhiwale R., Thomas V. (2007). Synthesis of Polymer Stabilized Silver and Gold Nanostructures. J. Nanosci. Nanotechnol..

[128] Jung S., Shuford K.L., Park S. (2011). Optical Property of a Colloidal Solution of Platinum and Palladium Nanorods: Localized Surface Plasmon Resonance. J. Phys. Chem. C.

[129] Wang Y., Liu X., Deng G., Wang Q., Zhang L., Wang Q., Lu J. (2017). Multifunctional PS@CS@Au-Fe_3_O_4_-FA Nanocomposites for CT, MR and Fluorescence Imaging Guided Targeted-Photothermal Therapy of Cancer Cells. J. Mater. Chem. B.

[130] Guo L., Yan D., Yang D., Li Y., Wang X., Zalewski O., Yan B., Lu W. (2014). Combinatorial Photothermal and Immuno Cancer Therapy using Chitosan-Coated Hollow Copper Sulfide Nanoparticles. ACS Nano.

[131] Wolfbeis O. (1997). Optical Fiber Sensors.

[132] Ueno T., Nagano T. (2011). Fluorescent Probes for Sensing and Imaging. Nat. Methods.

[133] Baranwal A., Kumar A., Priyadharshini A., Oggu G.S., Bhatnagar I., Srivastava A., Chandra P. (2018). Chitosan: An Undisputed Bio-Fabrication Material for Tissue Engineering and Bio-Sensing Applications. Int. J. Biol. Macromol..

[134] Bhatnagar I., Mahato K., Ealla K.K.R., Asthana A., Chandra P. (2018). Chitosan Stabilized Gold Nanoparticle Mediated Self-Assembled gliP Nanobiosensor for Diagnosis of Invasive Aspergillosis. Int. J. Biol. Macromol..

[135] Wang Y., Zhu J., Zhu R., Zhu Z., Lai Z., Chen Z. (2003). Chitosan/Prussian Blue-Based Biosensors. Meas. Sci. Technol..

[136] Xia H., He G., Peng J., Li W., Fang Y. (2010). Preparation and Fluorescent Sensing Applications of Novel CdSe–chitosan Hybrid Films. Appl. Surface Sci..

[137] Lee H., Kim M., Yoon Y., Park W. (2017). Fluorescent Property of Chitosan Oligomer and its Application as a Metal Ion Sensor. Mar. Drugs.

[138] Guan H., Yu J., Chi D. (2013). Label-Free Colorimetric Sensing of Melamine Based on Chitosan-Stabilized Gold Nanoparticles Probes. Food Control.

[139] Amanulla B., Palanisamy S., Chen S., Chiu T., Velusamy V., Hall J., Chen T., Ramaraj S. (2017). Selective Colorimetric Detection of Nitrite in Water using Chitosan Stabilized Gold Nanoparticles Decorated Reduced Graphene Oxide. Sci. Rep..

[140] Chen Z., Wang Z., Chen X., Xu H., Liu J. (2013). Chitosan-Capped Gold Nanoparticles for Selective and Colorimetric Sensing of Heparin. J. Nanopart. Res..

[141] Zhang L., Zheng W., Tang R., Wang N., Zhang W., Jiang X. (2016). Gene Regulation with Carbon-Based siRNA Conjugates for Cancer Therapy. Biomaterials.

[142] Choi C., Nam J., Nah J. (2016). Application of Chitosan and Chitosan Derivatives as Biomaterials. Ind. Eng. Chem. Res..

